# Hospitalizations and Mortality From Non–SARS-CoV-2 Causes Among Medicare Beneficiaries at US Hospitals During the SARS-CoV-2 Pandemic

**DOI:** 10.1001/jamanetworkopen.2022.1754

**Published:** 2022-03-09

**Authors:** Alexander Dang, Ravi Thakker, Shuang Li, Erin Hommel, Hemalkumar B. Mehta, James S. Goodwin

**Affiliations:** 1 Department of Medicine and the Sealy Center on Aging, The University of Texas Medical Branch at Galveston; 2 Department of Epidemiology, Johns Hopkins Bloomberg School of Public Health, Baltimore, Maryland

## Abstract

**Importance:**

The increased hospital mortality rates from non–SARS-CoV-2 causes during the SARS-CoV-2 pandemic are incompletely characterized.

**Objective:**

To describe changes in mortality rates after hospitalization for non–SARS-CoV-2 conditions during the COVID-19 pandemic and how mortality varies by characteristics of the admission and hospital.

**Design, Setting, and Participants:**

Retrospective cohort study from January 2019 through September 2021 using 100% of national Medicare claims, including 4626 US hospitals. Participants included 8 448 758 individuals with non–COVID-19 medical admissions with fee-for-service Medicare insurance.

**Main Outcomes and Measures:**

Outcome was mortality in the 30 days after admission with adjusted odds generated from a 3-level (admission, hospital, and county) logistic regression model that included diagnosis, demographic variables, comorbidities, hospital characteristics, and hospital prevalence of SARS-CoV-2.

**Results:**

There were 8 448 758 non–SARS-CoV-2 medical admissions in 2019 and from April 2020 to September 2021 (mean [SD] age, 73.66 [12.88] years; 52.82% women; 821 569 [11.87%] Black, 438 453 [6.34%] Hispanic, 5 351 956 [77.35%] White, and 307 218 [4.44%] categorized as other). Mortality in the 30 days after admission increased from 9.43% in 2019 to 11.48% from April 1, 2020, to March 31, 2021 (odds ratio [OR], 1.20; 95% CI, 1.19-1.21) in multilevel logistic regression analyses including admission and hospital characteristics. The increase in mortality was maintained throughout the first 18 months of the pandemic and varied by race and ethnicity (OR, 1.27; 95% CI, 1.23-1.30 for Black enrollees; OR, 1.25; 95% CI, 1.23-1.27 for Hispanic enrollees; and OR, 1.18; 95% CI, 1.17-1.19 for White enrollees); Medicaid eligibility (OR, 1.25; 95% CI, 1.24-1.27 for Medicaid eligible vs OR, 1.18; 95% CI, 1.16-1.18 for noneligible); and hospital quality score, measured on a scale of 1 to 5 stars with 1 being the worst and 5 being the best (OR, 1.27; 95% CI, 1.22-1.31 for 1 star vs OR, 1.11; 95% CI, 1.08-1.15 for 5 stars). Greater hospital prevalence of SARS-CoV-2 was associated with greater increases in odds of death from the prepandemic period to the pandemic period; for example, comparing mortality in October through December 2020 with October through December 2019, the OR was 1.44 (95% CI, 1.39-1.49) for hospitals in the top quartile of SARS-CoV-2 admissions vs an OR of 1.19 (95% CI, 1.16-1.22) for admissions to hospitals in the lowest quartile. This association was mostly limited to admissions with high-severity diagnoses.

**Conclusions and Relevance:**

The prolonged elevation in mortality rates after hospital admission in 2020 and 2021 for non–SARS-CoV-2 diagnoses contrasts with reports of improvement in hospital mortality during 2020 for SARS-CoV-2. The results of this cohort study suggest that, with the continued impact of SARS-CoV-2, it is important to implement interventions to improve access to high-quality hospital care for those with non–SARS-CoV-2 diseases.

## Key Points

**Question:**

Have outcomes of patients with non–SARS-CoV-2 illness changed during the COVID-19 pandemic?

**Findings:**

In this cohort study of 8 448 758 Medicare admissions to 4626 US hospitals from 2019 and April 2020 through September 2021, admissions for non–SARS-CoV-2 diagnoses fell sharply in March and April 2020 and remained lower through September 2021. Mortality rates after hospitalization were substantially higher, especially for Black individuals, Hispanic individuals, and those with low socioeconomic status, and the increases in mortality were greater in lower-quality hospitals and hospitals with high caseloads of SARS-CoV-2.

**Meaning:**

The prolonged increases in mortality rates after hospitalization for non–SARS-CoV-2 illnesses suggest a need for improved access to hospital care for individuals with non–SARS-CoV-2 illnesses.

## Teaser

This cohort study investigates how hospitalizations and mortality rates for non–SARS-CoV-2 conditions have changed during the COVID-19 pandemic.

## Introduction

The increase in all-cause mortality in the United States during the SARS-CoV-2 pandemic is not entirely explained by deaths from SARS-CoV-2 infection.^1,2^ Since the beginning of the pandemic, hospital admissions for non–SARS-CoV-2 diseases declined markedly and mortality rates increased.^3,4,5,6,7,8,9,10,11,12,13^ In the United States and other countries, areas with higher SARS-CoV-2 prevalence or higher proportions of low-income or racial and ethnic minority residents had larger increases in deaths from non–SARS-CoV-2 causes.^2,3,14^ Avoidance of emergency care by patients owing to concern for risk of SARS-CoV-2 infection, stay-at-home orders, decreases in surveillance, and other barriers to access to medical care most certainly contributed to excess deaths.^15,16,17,18,19^ Approximately 4 of 10 US adults delayed or avoided medical care by the end of June 2020 owing to concerns about SARS-CoV-2.^16^

Several groups have reported higher in-hospital mortality for patients with non–COVID-19 illness, with conflicting results whether this occurred only early in the pandemic or continued into early 2021.^3,20,21,22^ Of the existing reports, most have focused on in-hospital mortality as opposed to 30-day mortality,^3,20,21^ although 30-day mortality better reflects hospital quality of care.^23^

We sought to characterize patterns of mortality in the 30 days after hospital admission, comparing the 12 months of 2019 (prepandemic period) to the 18 months of April 1, 2020, through September 30, 2021 (pandemic period). We assessed whether changes in mortality varied by admission and hospital characteristics. We hypothesized that change in risk of death from the prepandemic to pandemic periods would be greater among disadvantaged patients, those with higher-severity diagnoses, and those admitted to lower-quality hospitals and to hospitals with a higher percentage of SARS-CoV-2 admissions.

## Methods

In this cohort study, we graphed the number of admissions for 20 common non–SARS-CoV-2 conditions at 4626 US acute care hospitals and their mortality rates for each month from January 2019 through September 2021. We compared changes in mortality 30 days after hospital admission during the pandemic with the prepandemic period using multilevel analyses adjusting for the contributions of admission and hospital characteristics and county. Because the pandemic changed over time, some analyses compared 3-month pandemic periods to the same months before the pandemic. We tested for interactions to assess whether the change in mortality over time varied by characteristics of the admission or hospital and performed stratified analyses where they were significant. We also assessed the association of hospital prevalence of SARS-CoV-2 with mortality for non–SARS-CoV-2 admissions, testing for interactions to determine whether the association varied by admission or hospital characteristics. The study was approved by The University of Texas Medical Branch institutional review board, which waived the need for informed consent because of the use of deidentified data. This study followed the Strengthening the Reporting of Observational Studies in Epidemiology (STROBE) reporting guidelines.^24^

### Data Sources

We used 100% of national Medicare claims from January 1, 2019, through October 31, 2021, in the analyses, last updated on December 15, 2021, including the Master Beneficiary Summary File and the Medicare Provider Utilization and Payment Data: Inpatient.

### Study Population

eFigure 1 in the Supplement shows the cohort selection flow. We included all acute hospitalizations between January 1, 2019, and September 30, 2021, then restricted the sample to those with Part A Medicare without health maintenance organization enrollment during hospitalization. We excluded patients with a SARS-CoV-2 admission diagnosis or SARS-CoV-2 in the first 2 discharge diagnoses, using *International Statistical Classification of Diseases and Related Health Problems, Tenth Revision (ICD-10)* code U07.1. For admissions in January to March 2020, we used a combination of codes J12.89, J20.8, J40, J22, J98.8, or J80 plus either codes B97.29 or U07.1.^25^ We restricted the admissions to those with 20 common medical diagnoses (eTable 1 in the Supplement) as defined by Birkmeyer et al.^3^

### Patient Characteristics

We extracted information on patient age, sex, race and ethnicity (Hispanic, non-Hispanic Black, non-Hispanic White, or other), and Medicaid eligibility from the Medicare Beneficiary Summary File. Race and ethnicity in Medicare is based on an algorithm using self-report and surname.^26^ Medicare records race and ethnicity as Asian, Black, Hispanic, North American Native, White, other, and unknown. We combined those in the Asian, North American Native, other, and unknown categories into “other.” The percentage of high school graduates in the patient’s zip code was obtained from the 2019 American Community Survey and categorized in quartiles.^27^ We used Elixhauser comorbidities based on the nonprimary diagnoses that were present on admission.^28^ Admission diagnosis, length of stay, and residence prior to hospitalization (community vs nursing facility/institution) were obtained from the inpatient file. In some analyses, we substituted a severity score for the 20 individual admitting diagnoses, generating the score based on probability of 30-day mortality for each diagnosis, using 2018 Medicare data (eTable 1 in the Supplement).

### Outcomes

We used the death date in Medicare Beneficiary Summary File to determine whether the patients died within 30 days after hospital admission.

### Hospital Characteristics

Information on hospital bed size, location (urban/rural), type (for profit, nonprofit, or public), and medical school affiliation (major, limited, graduate, or no affiliation) was extracted from the Provider of Service file 2020.^29^ We calculated the prevalence of SARS-CoV-2 for specified periods for each hospital, using the number of SARS-CoV-2 Medicare hospitalizations divided by the number of all Medicare hospitalizations in that period, and categorized into quartiles. If the prevalence was 0 in more than 25% of hospitals in a period, we categorized into terciles (0, >0 to 75th percentile, and >75th percentile). The 5-star quality rating for each hospital for 2019 was obtained from the overall hospital rating on the Hospital Consumer Assessment of Healthcare Providers and Systems survey.^30^

### Statistical Analyses

We graphed the numbers of hospitalizations and 30-day mortality rates for each month from January 1, 2019, to September 30, 2021. We constructed 3-level logistic regression (admission, hospital, and county) models to generate the odds of 30-day mortality after admission during April 1, 2020, to March 31, 2021, compared with 2019, adjusted for patient and hospital characteristics (see eMethods in the Supplement for details). We repeated these and subsequent analyses using 3-month cohorts, comparing the second, third, and fourth quarter of 2020 and the first, second, and third quarter of 2021 with the comparable quarters of 2019. We tested for interactions between time and either admission or hospital characteristics by adding the interaction terms to the model. We used *F* statistics for interaction terms and considered α less than .05 as significant. We conducted stratified analyses for significant interactions.

We investigated the association of 30-day mortality with hospital SARS-CoV-2 prevalence during six 3-month periods in April 1, 2020, through September 30, 2021. We included interaction terms between hospital SARS-CoV-2 prevalence and hospital and admission characteristics and performed stratified analyses where significant. In secondary analyses, we examined hospital mortality rather than mortality in the 30 days after hospital admission.

We conducted sensitivity analyses excluding admissions with SARS-CoV-2 infection using broader methods to define admissions with SARS-CoV-2, such as excluding those with a diagnosis of SARS-CoV-2 in the 30 days before or after hospitalization

 Statistical significance was defined as a 95% CI excluding 1 for ratios. Because of the very large number of admissions, almost all differences between groups were statistically significant. Thus, we focused on clinically important differences. All analyses were performed with SAS Enterprise, version 7.1 (SAS Institute Inc), at the Centers for Medicare & Medicaid Services Virtual Research Data Center.

## Results

### Trends in Admissions and Mortality Before and During the SARS-CoV-2 Pandemic

There were 8 448 758 non–SARS-CoV-2 medical admissions for 5 573 419 enrollees in all of 2019 and April 2020 through September 2021 (mean [SD] age, 73.66 [12.88] years; 52.82% women; 821 569 [11.87%] Black, 438 453 [6.34%] Hispanic, 5 351 956 [77.35%] White, and 307 218 [4.44%] categorized as other [including Asian, North American Native, and unknown]). The characteristics of the non–SARS-CoV-2 medical admissions in the 18 months from April 1, 2020, to September 30, 2021, differed somewhat from those in 2019, as did admission diagnoses (eTable 2 in the Supplement). For example, admissions during the pandemic had a lower percentage of Medicaid-eligible enrollees (26.7% vs 28.9%) and a higher percent admitted from nursing homes (12.9% vs 12.1%).The Figure shows the number of all admissions and non–SARS-CoV-2 medical admissions of fee-for-service Medicare patients to 4626 US acute care hospitals in each month from January 1, 2019, to September 30, 2021, and also the mortality rates in the 30 days after hospital admission. There was a steep decline in non–SARS-COV-2 medical admissions in March and April 2020. This was mirrored by an increase in mortality rates for non–SARS-CoV-2 medical admissions, which were between 8.9% and 10.2% during 2019 and increased to 12.4% in March and 13.5% in April 2020, followed by a second peak of more than 13% in November 2020 through January 2021, and a third increase in August and September 2021. eFigure 2 in the Supplement shows the monthly trend in mortality rates in each of the 9 census divisions. All geographic areas showed peaks in mortality during March through May 2020, October 2020 to January 2021, and August through September 2021, with different magnitudes. Also, the temporal patterns of admission and mortality were similar for each of the 20 admission diagnoses when graphed separately (eFigure 3 in the Supplement).

**Figure.**
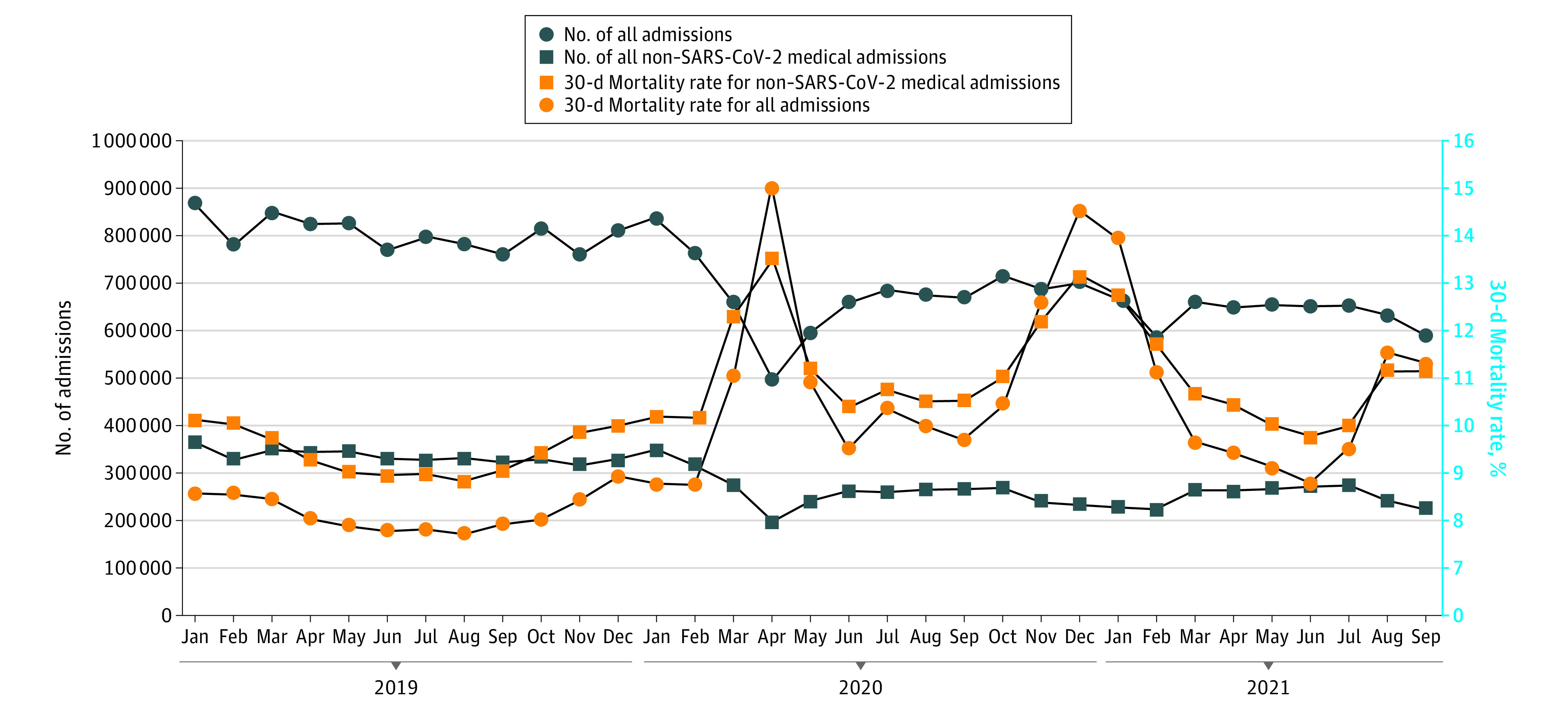
Number of Hospital Admissions and 30-Day Mortality Rates Following Hospitalization, by Month, from January 2019 Through September 2021 for US Fee-for-Service Medicare Enrollees Results for all admissions and for non–SARS-CoV-2 medical admissions are shown. There was a steep decline in non–SARS-CoV-2 medical admissions, from 370 007 and 355 898 in March and April 2019 to 281 383 and 200 679 in March and April 2020 (23.9% and 43.0% decreases, respectively). Mortality for non–SARS-CoV-2 medical admissions peaked at 13.46% in April 2020 and again in December (13.16%) and in August 2021 (11.19%).

### Association of the SARS-CoV-2 Pandemic With Mortality for Non–SARS-COV-2 Medical Admissions

Table 1 presents unadjusted mortality rates and results from a multilevel (admission, hospital, and county) logistic regression model comparing mortality in the 30 days after hospital admission during the initial 12 months of the pandemic period, April 2020 through March 2021, with the 12 months of 2019 for admissions with a non–SARS-COV-2 medical diagnosis. The regression model includes admission and hospital characteristics, including the 31 comorbidities listed in eTable 3 in the Supplement. Unadjusted mortality rates for non–SARS-CoV-2 medical admissions increased from 9.43% in the prepandemic period to 11.48% in the pandemic period. In the multilevel model, patients hospitalized during the pandemic were 20% more likely to die (OR, 1.20; 95% CI, 1.19-1.21) compared with the prepandemic period. Hospital mortality showed a similar pattern; 3.97% in 2019 vs 4.90% in April 1, 2020, through March 31, 2021 (OR, 1.16; 95% CI, 1.15-1.17) (eTable 4 in the Supplement).

**Table 1.** Mortality in the 30 Days After Hospital Admission for Non–SARS-CoV-2 Medical Diagnoses During January to December 2019 and April 2020 to March 2021, Unadjusted Rates and Odds Ratios From 3-Level (Admission, Hospital, and County) Logistic Regression Analysis^a^

Characteristic	No. (%)	30-d Mortality after admission, No. (%)	Odds ratio (95% CI)
All	6 919 196	712 708 (10.30)	NA
Period			
January 2019-December 2019	3 983 950 (57.58)	375 605 (9.43)	1 [Reference]
April 2020-March 2021	2 935 246 (42.42)	337 103 (11.48)	1.20 (1.19-1.21)
Length of stay, d	NA	NA	0.99 (0.98-0.99)
Age, y			
≤65	1 472 200 (21.28)	86 135 (5.85)	1 [Reference]
66-70	1 096 723 (15.85)	85 390 (7.79)	1.31 (1.29-1.32)
71-75	1 121 875 (16.21)	101 885 (9.08)	1.55 (1.53-1.56)
76-80	1 055 721 (15.26)	110 744 (10.49)	1.82 (1.80-1.84)
81-85	930 264 (13.44)	115 332 (12.40)	2.25 (2.23-2.28)
≥86	1 242 413 (17.96)	213, 222 (17.16)	3.50 (3.47-3.54)
Sex			
Male	3 266 768 (47.21)	355 252 (10.87)	1 [Reference]
Female	3 652 428 (52.79)	357 456 (9.79)	0.92 (0.92-0.93)
Race and ethnicity			
Black	821 569 (11.87)	76 770 (9.34)	0.91 (0.90-0.92)
Hispanic	438 453 (6.34)	41 651 (9.50)	0.99 (0.98-1.01)
White	5 351 956 (77.35)	563 510 (10.53)	1 [Reference]
Other^b^	307 218 (4.44)	30 777 (10.02)	0.97 (0.95-0.98)
Medicaid			
No	4 973 602 (71.88)	531 610 (10.69)	1 [Reference]
Yes	1 945 594 (28.12)	181 098 (9.31)	0.97 (0.97-0.99)
Education (persons aged ≥25 y in zip code area with a high school education), %			
Quartile 1	1 788 498 (25.85)	185 729 (10.38)	1 [Reference]
Quartile 2	1 761 020 (25.45)	183 089 (10.40)	0.99 (0.98-0.99)
Quartile 3	1 750 825 (25.30)	179 924 (10.28)	0.97 (0.96-0.97)
Quartile 4	1 618 853 (23.40)	169 966 (10.13)	0.94 (0.93-0.95)
Residence prior to hospitalization			
Community	6 060 688 (87.59)	573 261 (9.46)	1 [Reference]
Nursing facility or other institutions	858 508 (12.41)	139 447 (16.24)	1.72 (1.70-1.73)
Admission category			
Abdominal pain	843 966 (12.20)	51 887 (6.15)	1 [Reference]
AMI	239 076 (3.46)	25 908 (10.84)	2.05 (2.02-2.09)
Alcohol related	80 080 (1.16)	8107 (10.12)	2.07 (2.01-2.12)
Altered mental status	105 459 (1.52)	14 090 (13.36)	1.57 (1.53-1.60)
Arrythmia	466 700 (6.75)	51 079 (10.94)	1.86 (1.83-1.89)
Chest pain	505 765 (7.31)	20 934 (4.14)	0.85 (0.83-0.87)
CHF	323 536 (4.68)	34 382 (10.63)	1.28 (1.26-1.30)
COPD	237 393 (3.43)	13 165 (5.55)	1.17 (1.15-1.20)
Dehydration	604 055 (8.73)	68 536 (11.35)	1.28 (1.26-1.30)
Diabetes	160 147 (2.31)	8712 (5.44)	1.00 (0.97-1.02)
Gastrointestinal bleeding	378 139 (5.47)	30 896 (8.17)	1.15 (1.13-1.17)
Hip fracture	152 156 (2.20)	11 919 (7.83)	1.26 (1.23-1.29)
Pancreatitis	63 682 (3.53)	2250 (3.53)	0.73 (0.70-0.77)
Pneumonia	439 673 (6.35)	60 051 (13.66)	1.91 (1.89-1.94)
Respiratory failure	284 115 (4.11)	55 467 (19.52)	3.09 (3.05-3.14)
Seizure	94 404 (1.36)	6624 (7.02)	1.10 (1.07-1.13)
Sepsis and sepsis shock	805 579 (11.64)	167 418 (20.78)	2.69 (2.66-2.72)
Skin and soft tissue infection (cellulitis)	295 468 (4.27)	13 367 (4.52)	0.90 (0.89-0.92)
Stroke	514 512 (7.44)	514 512 (9.10)	1.23 (1.21-1.24)
UTI	325 291 (4.70)	21 076 (6.48)	0.80 (0.79-0.82)
Hospital characteristics			
Location			
Rural	907 417 (13.11)	96 911 (10.68)	1 [Reference]
Urban	6 011 779 (86.89)	615 797 (10.24)	0.85 (0.83-0.88)
Type of hospital			
For profit	1 037 227 (14.99)	104 666 (10.09)	1 [Reference]
Government	854 820 (12.35)	90 389 (10.57)	1.12 (1.08-1.16)
Nonprofit	5 027 149 (72.66)	517 653 (10.30)	0.98 (0.96-1.01)
Bed size, No.			
≤200	1 820 950 (26.32)	183 994 (10.10)	1 [Reference]
201-350	1 835 572 (26.53)	190 928 (10.40)	1.04 (1.01-1.07)
351-500	1 226 921 (17.73)	129 659 (10.57)	1.07 (1.03-1.11)
≥501	2 035 753 (29.42)	208 127 (10.22)	1.02 (0.99-1.06)
Medical school affiliation			
Major	1 610 211 (23.27)	162 340 (10.08)	1 [Reference]
Limited	1 503 721 (21.73)	155 740 (10.36)	1.03 (0.99-1.06)
Graduate	345 069 (4.99)	36 542 (10.59)	1.07 (1.01-1.13)
No affiliation	3 460 195 (50.01)	358 086 (10.35)	1.07 (1.03-1.10)
HCAHPS star rating			
1	156 679 (2.26)	16 773 (10.71)	1 [Reference]
2	896 157 (12.95)	94 449 (10.54)	0.99 (0.93-1.06)
3	3 460 499 (50.01)	360 135 (10.41)	0.95 (0.90-1.01)
4	1 984 041 (28.67)	200 131 (10.09)	0.91 (0.86-0.97)
5	237 628 (3.43)	22 077 (9.29)	0.90 (0.83-0.96)
Not available	184 192 (2.66)	19 143 (10.39)	1.12 (1.05-1.20)

Abbreviations: AMI, acute myocardial infarction; CHF, congestive heart failure; COPD, chronic obstructive pulmonary disease; HCAHPS, Hospital Consumer Assessment of Healthcare Providers and Systems; NA, not applicable; UTI, urinary tract infection.

^a^ The logistic regression model also includes 31 comorbidities shown in eTable 3 in the Supplement.

^b^ Medicare records race and ethnicity as Asian, Black, Hispanic, North American Native, White, other, and unknown. We combined those in the Asian, North American Native, other, and unknown categories into “other.”

We repeated those analyses using 6 periods: April to June, July to September, and October to December for 2020 and January to March, April to June, and July to September for 2021, each compared with the same months in 2019. The odds of 30-day mortality in the pandemic vs prepandemic periods were 1.23 (95% CI, 1.21-1.24) in April to June 2020, 1.17 (95% CI, 1.16-1.18) in July to September 2020, 1.28 (95% CI, 1.26-1.29) in October to December 2020, 1.13 (95% CI, 1.12-1.14) for January to March 2021, 1.06 (95% CI, 1.05-1.07) for April to June, and 1.17 (95% CI, 1.16-1.18) for July to September 2021 (Table 2).

**Table 2.** Unadjusted 30-Day Mortality Rates and Odds Ratios After Admission for a Non–SARS-CoV-2 Medical Condition in April 2020 Through September 2021, Compared With 2019, Stratified by 3-Month Periods^a^

Variable	Odds ratio (95% CI)					
	April-June 2020 vs April-June 2019	July-September 2020 vs July-September 2019	October-December 2020 vs October-December 2019	January-March 2021 vs January-March 2019	April-June 2021 vs April-June 2019	July-September 2021 vs July-September 2019
2020 vs 2019 (Unadjusted rates), %	11.55 vs 9.11	10.58 vs 8.96	12.09 vs 9.64	NA	NA	NA
2019	1 [Reference]	1 [Reference]	1 [Reference]	NA	NA	NA
2020	1.23 (1.21-1.24)	1.17 (1.16-1.18)	1.28 (1.26-1.29)	NA	NA	NA
2021 vs 2019 (Unadjusted rates), %	NA	NA	NA	11.78 vs 9.98	10.09 vs 9.11	10.75 vs 8.96
2019	NA	NA	NA	1 [Reference]	1 [Reference]	1 [Reference]
2021	NA	NA	NA	1.13 (1.12-1.14)	1.06 (1.05-1.07)	1.17 (1.16-1.18)

Abbreviation: NA, not applicable.

^a^ Odds are from a 3-level (admission, hospital, and county) logistic regression analysis identical to the analysis presented in Table 1. All the variables included in the analyses in Table 1 are also included in the model but are not presented.

### Association of Admission and Hospital Characteristics With Mortality for Non–SARS-CoV-2 Medical Admissions

Table 3 presents the odds of death in the first 12 months of the pandemic (April 1, 2020, through March 31, 2021) vs 2019, stratified by the admission and hospital characteristics for which there were significant interactions with time in the analyses in Table 1. Black patients (OR, 1.27; 95% CI, 1.23-1.30) and Hispanic patients (OR, 1.25; 95% CI, 1.23-1.27) experienced larger increases in odds of death from the prepandemic to pandemic periods than did White patients (OR, 1.18; 95% CI, 1.17-1.19). The increases in 30-day mortality were also larger among Medicaid-eligible admissions (OR, 1.25; 95% CI, 1.24-1.27 for Medicaid vs OR, 1.18; 95% CI, 1.16-1.18 for noneligible) and those admitted from the community rather than nursing homes (OR, 1.21; 95% CI, 1.19-1.21 for community vs OR, 1.15; 95% CI 1.14-1.16 for nursing home residents). There was also a substantial increase in odds of death from the prepandemic period to the pandemic period with decreasing hospital quality ratings, from OR, 1.11 (95% CI, 1.08-1.15) for admissions to 5-star hospitals to OR, 1.27 (95% CI, 1.22-1.31) for 1-star hospitals. Change in mortality from the prepandemic period to the pandemic period also varied by admitting diagnosis. Admissions for pneumonia (OR, 1.42; 95% CI, 1.39-1.45), cellulitis (OR, 1.28; 95% CI, 1.24-1.33), and urinary tract infection (OR, 1.26; 95% CI, 1.22-1.29) had the highest mortality increases, while admissions with alcohol-related diagnoses (OR, 0.99; 95% CI, 0.94-1.04) experienced no increase.

**Table 3.** Mortality in the 30 Days After Hospital Admission for Non–SARS-CoV-2 Medical Diagnoses in 2019 and in April 2020 to March 2021, With Adjusted Odds of Death, Comparing the 2 Periods Stratified by Characteristics of the Admission and Hospital^a^

Admission characteristics	No. of admissions (30-d mortality rate)		April 2020 to March 2021 vs January 2019 to December 2019, OR (95% CI)
	January 2019 to December 2019	April 2020 to March 2021	
Age, y			
≤65	858 813 (5.30)	613 387 (6.63)	1.19 (1.17-1.21)
66-70	624 961 (7.09)	471 762 (8.71)	1.17 (1.15-1.19)
71-75	636 165 (8.21)	485 710 (10.23)	1.20 (1.19-1.22)
76-80	602 157 (9.51)	453 564 (11.80)	1.21 (1.19-1.22)
81-85	533 399 (11.36)	396 865 (13.80)	1.20 (1.18-1.21)
≥86	728 455 (15.89)	513 958 (18.96)	1.20 (1.19-1.21)
Race and ethnicity			
Black	476 762 (8.28)	344 807 (10.82)	1.27 (1.23-1.30)
Hispanic	256 040 (8.42)	182 413 (11.01)	1.25 (1.23-1.27)
White	3 077 794 (9.72)	2 274 162 (11.63)	1.18 (1.17-1.19)
Other^b^	173 354 (8.94)	133 864 (11.41)	1.28 (1.25-1.31)
Medicaid			
No	2 833 520 (9.86)	2 140 082 (11.78)	1.18 (1.16-1.18)
Yes	1 150 430 (8.36)	795 164 (10.68)	1.25 (1.24. 1.27)
Education (% of persons aged ≥25 y in zip area with high school education), per %			
Quartile 1	1 048 242 (9.34)	740 256 (11.86)	1.25 (1.24-1.26)
Quartile 2	1 016 381 (9.51)	744 639 (11.61)	1.20 (1.18-1.21)
Quartile 3	1 001 517 (9.48)	749 308 (11.34)	1.17 (1.16-1.18)
Quartile 4	917 810 (9.38)	701 043 (11.11)	1.17 (1.16-1.18)
Residence prior to hospitalization			
Community	3 502 381 (8.63)	2 558 307 (10.60)	1.21 (1.19-1.21)
Nursing facility or other institutions	481 569 (15.25)	376 939 (17.51)	1.15 (1.14-1.16)
Admission diagnosis			
Abdominal pain	489 870 (5.76)	354 096 (6.69)	1.14 (1.12-1.16)
AMI	139 581 (10.38)	109 495 (11.38)	1.10 (1.07-1.13)
Alcohol related	45 575 (9.97)	34 505 (10.32)	0.99 (0.94-1.04)
Altered mental status	55 781 (12.38)	49 678 (14.46)	1.18 (1.13-1.22)
Arrythmia	272 393 (10.12)	194 307 (12.11)	1.17 (1.15-1.20)
Chest pain	300 849 (3.90)	204 916 (4.49)	1.14 (1.10-1.17)
CHF	190 264 (10.18)	133 272 (11.27)	1.13 (1.10-1.15)
COPD	167 997 (5.14)	69 396 (6.52)	1.24 (1.20-1.29)
Dehydration	329 931 (10.49)	274 124 (12.37)	1.18 (1.16-1.20)
Diabetes	89 028 (4.93)	71 119 (6.07)	1.20 (1.15-1.26)
Gastrointestinal bleeding	211 469 (7.52)	166 670 (9.00)	1.17 (1.14-1.19)
Hip fracture	78 713 (7.40)	73 443 (8.30)	1.10 (1.06-1.15)
Pancreatitis	36 201 (3.17)	27 481 (4.02)	1.24 (1.14-1.35)
Pneumonia	280 393 (12.01)	159 280 (16.56)	1.42 (1.39-1.45)
Respiratory failure	160 736 (18.14)	123 379 (21.33)	1.19 (1.17-1.21)
Seizure	52 289 (6.42)	42 115 (7.76)	1.14 (1.08-1.20)
Sepsis and sepsis shock	446 417 (19.08)	359 162 (22.90)	1.23 (1.21-1.24)
Skin and soft tissue infection (cellulitis)	170 649 (3.99)	124 819 (5.26)	1.28 (1.24-1.33)
Stroke	289 497 (8.41)	225 015 (9.99)	1.16 (1.13-1.18)
UTI	186 317 (5.80)	138 974 (7.38)	1.26 (1.22-1.29)
Hospital characteristics			
Location			
Rural	534 336 (9.79)	373 081 (11.96)	1.23 (1.21-1.24)
Urban	3 449 614 (9.37)	2 562 165 (11.42)	1.19 (1.18-1.20)
Type of hospital			
For profit	604 031 (9.07)	433 196 (11.52)	1.20 (1.19-1.22)
Government	489 644 (9.62)	365 176 (11.86)	1.22 (1.20-1.24)
Nonprofit	2 890 275 (9.47)	2 136 874 (11.41)	1.19 (1.18-1.20)
Bed size			
≤200	1 057 760 (9.20)	763 190 (11.36)	1.23 (1.22-1.24)
201-350	1 056 747 (9.53)	778 825 (11.58)	1.19 (1.17-1.20)
351-500	708 779 (9.65)	518 142 (11.83)	1.21 (1.20-1.23)
≥501	1 160 664 (9.40)	875 089 (11.31)	1.17 (1.16-1.18)
Medical school affiliation			
Major	920 634 (9.31)	689 577 (11.12)	1.17 (1.16-1.18)
Limited	867 518 (9.48)	636 203 (11.55)	1.19 (1.18-1.20)
Graduate	198 722 (9.73)	146 347 (11.76)	1.17 (1.15-1.20)
No affiliation	1 997 076 (9.43)	1 463 119 (11.60)	1.21 (1.20-1.22)
HCAHPS star rating^c^			
1	91 925 (9.52)	64 754 (12.38)	1.27 (1.22-1.31)
2	526 636 (9.49)	369 521 (12.04)	1.23 (1.21-1.25)
3	1 995 431 (9.50)	1 465 038 (11.64)	1.20 (1.19-1.21)
4	1 130 239 (9.34)	853 802 (11.07)	1.17 (1.16-1.18)
5	132 647 (8.76)	104 981 (9.96)	1.11 (1.08-1.15)

Abbreviations: AMI, acute myocardial infarction; CHF, congestive heart failure; COPD, chronic obstructive pulmonary disease; HCAHPS, Hospital Consumer Assessment of Healthcare Providers and Systems; OR, odds ratio; UTI, urinary tract infection.

^a^ Only admission characteristics that had a significant interaction with time (April 2020 to March 2021 vs January 2019 to December 2019) in the analyses shown in Table 1 are presented in this table. For each characteristic, we conducted a separate 3-level logistic regression analysis, as in Table 1, and included all the admission characteristics.

^b^ Medicare records race and ethnicity as Asian, Black, Hispanic, North American Native, White, other, and unknown. We combined those in the Asian, North American Native, other, and unknown categories into “other.”

^c^ Hospitals with 184 192 admissions did not have Centers for Medicare & Medicaid Services quality ratings. They were included in the analysis as a separate category but not shown.

### Association of Hospital SARS-CoV-2 Prevalence With Mortality for Non–SARS-CoV-2 Admissions

Table 4 presents the mortality rates and adjusted odds of death during the pandemic compared with the same months in 2019, stratified by whether the hospital had a high or low prevalence of SARS-CoV-2 cases. We divided the pandemic into six 3-month periods because hospital prevalence of SARS-CoV-2 changed throughout the pandemic. In each period, hospitals with high SARS-CoV-2 prevalence experienced substantially greater increases in mortality for non–SARS-CoV-2 admissions than did hospitals with lower prevalence. For example, comparing October to December 2020 vs October to December 2019, the OR for mortality was 1.44 (95% CI, 1.39-1.49) for admissions to high-prevalence hospitals vs OR, 1.19 (95% CI, 1.16-1.22) for low-prevalence hospitals.

**Table 4.** Changes in Mortality for Non–SARS-CoV-2 Admissions From the Prepandemic Period to the Pandemic Period, Stratified by Hospital Prevalence of SARS-CoV-2 Cases^a^

Time	Hospital SARS-CoV-2 prevalence (%)^b^	Mortality rate, %		OR (95% CI)^a^
		2019	2020-2021	
April-June 2019 and 2020	High (>2.06)	9.15	12.38	1.34 (1.32-1.36)
	Low (0)	8.82	10.42	1.17 (1.13-1.21)
July-September 2019 and 2020	High (>3.94)	9.56	12.13	1.26 (1.23-1.29)
	Low (0)	8.00	9.05	1.15 (1.10-1.20)
October-December 2019 and 2020	High (>14.97)	10.19	13.92	1.44 (1.39-1.49)
	Low (<2.87)	9.05	10.66	1.19 (1.16-1.22)
January-March 2019 and 2021	High (>8.66)	10.30	13.31	1.28 (1.24-1.32)
	Low (<1.61)	9.57	10.57	1.09 (1.05-1.12)
April-June 2019 and 2021	High (>2.12)	9.23	10.70	1.13 (1.11-1.16)
	Low (0)	8.39	9.32	1.11 (1.06-1.16)
July-September 2019 and 2021	High (>9.38)	10.81	12.68	1.37 (1.32-1.41)
	Low (<1.46)	8.21	9.26	1.12 (1.09-1.15)

Abbreviation: OR, odds ratio.

^a^ The results are generated in multilevel (admission, hospital, and county) logistic regression models containing all admission and hospital characteristics in Table 1; eTable 3 in the Supplement.

^b^ For each period, the hospitals were ranked by percentage of all Medicare admissions that were for SARS-CoV-2. Hospital SARS-CoV-2 prevalence was grouped by quartile. In periods where the prevalence of SARS-CoV-2 was 0 for greater than 25% of hospitals (44.49% for April-June 2020; 30.75% for July-September 2020; and 31.67% for April-June 2021), we grouped into 3 groups (0, >0 to <75th percentile, and ≥75th percentile).

We next examined whether the association between the hospital prevalence of SARS-CoV-2 and mortality varied by the characteristics of the admissions or hospitals. We tested for interactions between SARS-CoV-2 prevalence and the admission and hospital characteristics and performed stratified analyses where the interactions were significant (eTables 5-10 in the Supplement). There were no consistent findings over the 6 periods, although in the first 5 periods, covering April 2020 through March 2021, the association between high hospital SARS-CoV-2 prevalence and increased odds of death was limited to admission diagnoses with a high expected mortality.

### Sensitivity Analysis

We conducted sensitivity analyses excluding admissions with SARS-CoV-2 infection using different definitions. In analyses with those cohorts, the increases in odds of death in 2020 vs 2019 were similar to those in the main analyses (eTable 11 in the Supplement).

## Discussion

In this cohort study, among fee-for-service Medicare enrollees, there was a steep decline in non–SARS-CoV-2 medical admissions beginning March 2020, which remained depressed through September of 2021. This was mirrored by an increase in 30-day and hospital mortality for non–SARS-CoV-2 medical admissions over the same period. Black and Hispanic patients had larger increases in mortality, as did those who were eligible for Medicaid, lived in zip codes associated with low education levels, or were admitted to lower-quality hospitals or hospitals with a higher prevalence of SARS-COV-2 cases.

Our analyses add to prior reports of increases in hospital mortality for non–SARS-CoV-2 admissions.^3,20,21,31^ In a study of 201 hospitals, Birkmeyer et al.^3^ reported an increase in non–SARS-COV-2 hospital mortality from 2.1% in February 2020 to 2.4% in April, returning to baseline in May and June 2020. We found larger increases in hospital mortality and in mortality in the 30 days after hospital admission, which were maintained to September of 2021. Compared with the same months in 2019, the odds of death were 17% to 28% higher in the final 3 quarters of 2021, fell to 6% higher in the April to June quarter of 2021, and were 17% higher in July to September 2021. The elevated mortality for non–SARS-CoV-2 admissions throughout the pandemic contrasts somewhat with reports on the pattern of mortality for SARS-CoV-2 admissions, which showed a downward trend over time.^32,33^

There are 2 main postulated mechanisms for the higher mortality. One posits that during the pandemic, those who were hospitalized tended to have more severe disease and higher risk of death. Delays in seeking care because of fear of exposure to SARS-CoV-2 or because of barriers to access to outpatient and emergency care during the pandemic would result in patients admitted sicker and later in their illness.^9,16,17,18,19,34,35,36,37^ A second possible mechanism is that a lack of critical hospital resources such as intensive care unit beds and personnel because of the hospitalized patients with SARS-CoV-2 resulted in lower-quality care for all patients.^34^ This latter possible mechanism is supported by greater mortality increases in rural hospitals, smaller hospitals, and hospitals that were not affiliated with medical schools during the pandemic compared with the prepandemic period.^38^ Also, mortality for non–SARS-CoV-2 illness during the pandemic was worse even after controlling for severity of illness.^37^

It was not possible in this study, nor would it be in any study using only administrative data, to determine the relative contributions of those 2 mechanisms to the excess mortality. Commonly used indicators of illness severity available in administrative data, such as length of stay or intensive care unit transfers, are not valid because they were affected by pandemic-related hospital crowding and would thus contribute to artifactually low estimates of disease severity.

Increases in risk of death in 2020 from non–SARS-COV-2 illnesses were associated with higher prevalence of SARS-COV-2 in the hospital, but only in those with more severe disease. Kadri et al^33^ reported a similar association among patients with SARS-COV-2. High severity would be more likely to require intensive care, a resource that was limited in hospitals caring for high levels of patients with SARS-CoV-2.

Studies from the United States and abroad have reported larger increases in SARS-CoV-2 all-cause mortality and hospital deaths during the pandemic among racial and ethnic minority individuals and those with low income.^2,3,21,35,36^ Impaired access to health care caused by the pandemic hit hardest at populations already experiencing disparities in health care access.^39,40^ For example, Cronin and Evans^1^ estimated that non-Hispanic Black men, 6.9% of the population, accounted for 28% of the 2020 excess deaths from non–SARS-CoV-2 cases.

### Limitations

The study has several limitations. First, the findings cannot be generalized to residents without Medicare or those with Medicare Advantage. While Medicare Advantage enrollees used to differ from fee-for-service enrollees in terms of socioeconomic status and comorbidities, this is no longer the case.^42^ In 2020, 36% of Medicare beneficiaries were enrolled in a Medicare Advantage plan, and the percentage varies widely across states.^41^ In addition, Medicare enrollees younger than 65 years are disabled or have end-stage kidney disease and are not representative of their age group. Second, we did not evaluate postacute care, which was disrupted during the pandemic and could significantly influence 30-day mortality. Third, part of the increase in mortality rates during the pandemic might be a result of undiagnosed SARS-CoV-2 in the hospitalized patients. However, the increase was sustained after diagnostic testing became more sensitive, and many of the largest increases in mortality were seen in diseases without respiratory presentations such as pancreatitis, diabetes, and cellulitis. Fourth, we did not study the role of shortages of essential resources, such as intensive care unit beds and medical staff, that may explain the increase in non–SARS-CoV-2 mortality rates. Fifth, we were not able to assess how much of the increased mortality was associated with selection for admissions with more acute and more advanced disease vs deficiencies in care by hospitals overwhelmed by patients with SARS-CoV-2.

## Conclusions

The COVID-19 pandemic is a major stress test for US hospitals. The association of pandemic-associated increases in mortality rates for non–SARS-CoV-2 illnesses with hospital quality scores was stronger than seen in studies linking quality scores to other outcomes. While the future is not predictable, the ongoing emergence of new SARS-CoV-2 variants may lead to a more prolonged pandemic. Because of this, patients with non–SARS-CoV-2 illnesses may continue to delay or avoid care, resulting in continued excess morbidity and mortality. Given the uncertain trajectory of the pandemic, health care clinicians and policy makers should develop strategies to provide optimal care to patients for non–SARS-CoV-2 illnesses, especially among racial and ethnic minority and socioeconomically disadvantaged patients. Health systems should monitor both hospital admissions and mortality in these groups, using prepandemic levels as a comparison. These metrics can then be used to monitor the results of interventions to improve access for those with serious non–SARS-CoV-2 illness during the continued pandemic.

## Notes

Supplement.
eMethods.
**eFigure 1.** Cohort Selection **eFigure 2.** Mortality Rate in the 30 Days After Hospital Admission for a Medical Diagnosis From 1/1/19 to 9/30/21, Stratified by Census Division **eFigure 3.** Mortality Rates in the 30 Days After Admission From 1/1/19 To 9/30/21, Stratified by Admitting Diagnosis **eTable 1.** Unadjusted and Adjusted 30-Day Mortality Rates for Each of the 20 Medical Admission Diagnoses, From 2018 Cohort **eTable 2.** Characteristics of Hospital Non–SARS-CoV-2 Medical Admissions in 2019 and in April 2020-March 2021, and 30-Day Mortality Rates **eTable 3.** Comorbidities Included in the Multilevel Logistic Regression Model Presented in Table 1, Along With Unadjusted Mortality Rates and Adjusted Odds of Mortality **eTable 4.** Hospital Mortality for Non–SARS-COV-2 Medical Admissions During April 2020 Through March 2021, and 2019, Unadjusted Rates and Odds From Three-Level (Admission, Hospital, County) Logistic Regression Analysis **eTable 5.** Odds of Mortality in the 30 Days After Hospital Admission for Patients With Non–SARS-CoV-2 Medical Diagnoses From April Through June of 2020 by Level of Prevalence of SARS-CoV-2 in the Hospital, Stratified by Characteristics of the Admission **eTable 6.** Odds of Mortality in the 30 Days After Hospital Admission for Patients With Non–SARS-CoV-2 Medical Diagnoses From July Through September of 2020 by Level of Prevalence of SARS-CoV-2 in the Hospital, Stratified by Characteristics of the Admission **eTable 7.** Odds of Mortality in the 30 Days After Hospital Admission for Patients With Non–SARS-CoV-2 Medical Conditions From October Through December of 2020 by Level of Prevalence of SARS-CoV-2 in the Hospital, Stratified by Characteristics of the Admission **eTable 8.** Odds of Mortality in the 30 Days After Hospital Admission From January Through March 2021 for Patients With Non–SARS-CoV-2 Medical Conditions, by Level of Prevalence of SARS-CoV-2 in the Hospital, Stratified by Characteristics of the Admission **eTable 9.** Odds of Mortality in the 30 Days After Hospital Admission for Patients With Non–SARS-CoV-2 Medical Conditions From April Through June 2020, by Level of Prevalence of SARS-CoV-2 in the Hospital, Stratified by Characteristics of the Admission **eTable 10.** Odds of Mortality in the 30 Days After Hospital Admission From July Through September 2021 for Patients With Non–SARS-CoV-2 Medical Conditions, by Level of Prevalence of SARS-CoV-2 in the Hospital, Stratified by Characteristics of the Admission **eTable 11.** Unadjusted Rates and Adjusted Odds of Mortality After Hospitalization for Non–SARS-CoV-2 Medical Admissions in 2020 vs 2019, Using Different Methods to Exclude SARS-CoV-2 Cases
