# Supplementary material for: Hospitalizations and Mortality From Non–SARS-CoV-2 Causes Among Medicare Beneficiaries at US Hospitals During the SARS-CoV-2 Pandemic
Source: JAMA Netw Open. 2022 Mar 9;5(3):e221754. doi: 10.1001/jamanetworkopen.2022.1754 (PMC8908076; doi:10.1001/jamanetworkopen.2022.1754)
Supplement: Supplement. — eMethods. eFigure 1. Cohort Selection eFigure 2. Mortality Rate in the 30 Days After Hospital Admission for a Medical Diagnosis From 1/1/19 to 9/30/21, Stratified by Census Division eFigure 3. Mortality Rates in the 30 Days After Admission From 1/1/19 To 9/30/21, Stratified by Admitting Diagnosis eTable 1. Unadjusted and Adjusted 30-Day Mortality Rates for Each of the 20 Medical Admission Diagnoses, From 2018 Cohort eTable 2. Characteristics of Hospital Non–SARS-CoV-2 Medical Admissions in 2019 and in April 2020-March 2021, and 30-Day Mortality Rates eTable 3. Comorbidities Included in the Multilevel Logistic Regression Model Presented in Table 1, Along With Unadjusted Mortality Rates and Adjusted Odds of Mortality eTable 4. Hospital Mortality for Non–SARS-COV-2 Medical Admissions During April 2020 Through March 2021, and 2019, Unadjusted Rates and Odds From Three-Level (Admission, Hospital, County) Logistic Regression Analysis eTable 5. Odds of Mortality in the 30 Days After Hospital Admission for Patients With Non–SARS-CoV-2 Medical Diagnoses From April Through June of 2020 by Level of Prevalence of SARS-CoV-2 in the Hospital, Stratified by Characteristics of the Admission eTable 6. Odds of Mortality in the 30 Days After Hospital Admission for Patients With Non–SARS-CoV-2 Medical Diagnoses From July Through September of 2020 by Level of Prevalence of SARS-CoV-2 in the Hospital, Stratified by Characteristics of the Admission eTable 7. Odds of Mortality in the 30 Days After Hospital Admission for Patients With Non–SARS-CoV-2 Medical Conditions From October Through December of 2020 by Level of Prevalence of SARS-CoV-2 in the Hospital, Stratified by Characteristics of the Admission eTable 8. Odds of Mortality in the 30 Days After Hospital Admission From January Through March 2021 for Patients With Non–SARS-CoV-2 Medical Conditions, by Level of Prevalence of SARS-CoV-2 in the Hospital, Stratified by Characteristics of the Admission eTable 9. Odds of Mortality in the 30 [file jamanetwopen-e221754-s001.pdf]

Supplementary Online Content

Dang A, Thakker R, Li S, Hommel E, Mehta HB, Goodwin JS. Hospitalizations and mortality from non–SARS-CoV-2 causes among Medicare beneficiaries at US hospitals during the SARS-CoV-2 pandemic. *JAMA Netw Open.* 2022;5(3):e221754.

doi:10.1001/jamanetworkopen.2022.1754

eMethods.
eFigure 1. Cohort Selection
eFigure 2. Mortality Rate in the 30 Days After Hospital Admission for a Medical Diagnosis From 1/1/19 to 9/30/21, Stratified by Census Division
eFigure 3. Mortality Rates in the 30 Days After Admission From 1/1/19 To 9/30/21, Stratified by Admitting Diagnosis
eTable 1. Unadjusted and Adjusted 30-Day Mortality Rates for Each of the 20 Medical Admission Diagnoses, From 2018 Cohort
eTable 2. Characteristics of Hospital Non–SARS-CoV-2 Medical Admissions in 2019 and in April 2020-March 2021, and 30-Day Mortality Rates
eTable 3. Comorbidities Included in the Multilevel Logistic Regression Model Presented in Table 1, Along With Unadjusted Mortality Rates and Adjusted Odds of Mortality
eTable 4. Hospital Mortality for Non–SARS-COV-2 Medical Admissions During April 2020 Through March 2021, and 2019, Unadjusted Rates and Odds From Three-Level (Admission, Hospital, County) Logistic Regression Analysis
eTable 5. Odds of Mortality in the 30 Days After Hospital Admission for Patients With Non–SARS-CoV-2 Medical Diagnoses From April Through June of 2020 by Level of Prevalence of SARS-CoV-2 in the Hospital, Stratified by Characteristics of the Admission
eTable 6. Odds of Mortality in the 30 Days After Hospital Admission for Patients With Non–SARS-CoV-2 Medical Diagnoses From July Through September of 2020 by Level of Prevalence of SARS-CoV-2 in the Hospital, Stratified by Characteristics of the Admission
eTable 7. Odds of Mortality in the 30 Days After Hospital Admission for Patients With Non–SARS-CoV-2 Medical Conditions From October Through December of 2020 by Level of Prevalence of SARS-CoV-2 in the Hospital, Stratified by Characteristics of the Admission
eTable 8. Odds of Mortality in the 30 Days After Hospital Admission From January Through March 2021 for Patients With Non–SARS-CoV-2 Medical Conditions, by Level of Prevalence of SARS-CoV-2 in the Hospital, Stratified by Characteristics of the Admission
eTable 9. Odds of Mortality in the 30 Days After Hospital Admission for Patients With Non–SARS-CoV-2 Medical Conditions From April Through June 2020, by Level of Prevalence of SARS-CoV-2 in the Hospital, Stratified by Characteristics of the Admission
eTable 10. Odds of Mortality in the 30 Days After Hospital Admission From July Through September 2021 for Patients With Non–SARS-CoV-2 Medical Conditions, by Level of Prevalence of SARS-CoV-2 in the Hospital, Stratified by Characteristics of the Admission
eTable 11. Unadjusted Rates and Adjusted Odds of Mortality After Hospitalization for Non–SARS-CoV-2 Medical Admissions in 2020 vs 2019, Using Different Methods to Exclude SARS-CoV-2 Cases

© 2022 Dang A et al. *JAMA Network Open.*

eMethods

We performed three-level hierarchical logistic regression models for the analyses. The main model structure included admissions (level 1), hospital (level 2) and county (level 3). We treated admissions as independent and identically distributed in the model. Patient characteristics were included as level 1 predictors. Hospital was level 2, with admissions clustered into hospitals. Hospital characteristics were included as level 2 predictors. Hospitals were clustered into counties, for level 3.

The main three-level model (Table 1) included random intercepts at level 2 (hospitals) and level 3 (counties). Time period, admission diagnosis and all other patient's characteristics were fixed effects at level 1, and all hospital characteristics were fixed effects in level 2. We chose the variance components (VC) for variance structure for both level 2 and level 3 random effects. Residual pseudo-likelihood estimation technique (RSPL) was used in the analysis.

The hierarchical structure included about 7M admissions, 4,600 hospitals and 2,500 counties. We chose the statistical estimation method based on the fit statistics, including Likelihood (-2 log Likelihood score); the ratio of Pearson or Generalized Chi–square; and the degree of freedom to examine the residual over-dispersion or under-dispersion. The computer time and memory capacity at the CMS Virtual Research Data Center were also considered in the analysis. At first, we tried to use the Maximum Likelihood Estimation based on the Adaptive Quadrature method, which reached the resource maximum limit at a small integration point (point=1,3,5). We abandoned this method because of insufficient resources for this procedure.

© 2022 Dang A et al. *JAMA Network Open.*

After that, we tested the Residual Pseudolikelihood Estimation Technique (RSPL) and maximum likelihood estimation with Laplace approximation (LAPLACE) for the model. We chose VC for the variance structure for level 2 and level 3 random intercepts. These methods provide a closed form solution to estimate the parameters, which make the computation faster. Both methods have similar estimation and fit statistics. However, RSPL takes about 1 hour per model, while LAPLACE requires approximately 16 hours per model. We chose RSPL for the analysis.

We performed the same main three-level logistic regression analyses for different cohorts, including the cohort in 2019 and April 2020 to March 2021 in Table 1, and the 6 quarterly cohorts in Table 2. All models included the same fixed effects and the same random effects. We further included interaction terms between time period and patient/hospital characteristics into the models. For Table 3, we added an interaction term for each variable into the model for Table 1. We checked the F statistics for the type 3 test of fixed effects for the interaction term. If the p-value was <0.05, we considered that interaction term significant. For significant interaction terms, we further performed stratified analyses by stratifying the cohort into sub-cohorts by that stratification variable. We then re-performed the same main model and reported the odd ratios (95% CI) for each sub-cohort in Table 3. For Table 4, we stratified the 6 quarterly cohorts into sub-cohorts by hospital SARS-CoV-2 prevalence during that quarter. Hospital SARS-CoV-2 prevalence was categorized by quartiles as a hospital-level variable. However, if >25% of hospitals in a time period had prevalence, we categorized the prevalence into 3 groups. We performed the three-level logistic regression model for the sub-cohorts (high

© 2022 Dang A et al. *JAMA Network Open.*

prevalence vs. low prevalence) hospitals for each quarterly cohort separately and reported odd ratios (95% CI) in Table 4.

The SAS code for the main analysis is below (Table 1). We initially tried the quad method (QPOINTS=X). We abandoned this method because of insufficient memory capacity. We then compared the LAPLACE method and the default RSPL method; both introduced similar estimations. However, the LAPLACE approximation takes about 16 hours computing time and the RSPL takes about 1 hour, so we choose the RSPL technique for our analysis.

```
proc glimmix data=Bir_ana;
 class  county_HOS prvdr_num y_treat (ref="1") severity_g1(ref="1")
 age_g(ref="1") BENE_SEX_IDENT_CD (ref="1") RACE_CD_u(ref="1")
 medicaid(ref="0") hsh_g(ref="1")  admit_from (ref='community')
 icd_g (ref="g1_abd")
 Alcohol_freq (ref="0")  Arrhy_freq  (ref="0")  BLA_freq(ref="0")
 CHF_freq(ref="0")   COPD_freq(ref="0")
 Coag_freq(ref="0")      DA_freq (ref="0")     Dep_freq(ref="0")  Diab_C_freq(ref="0")  Diab_UC_freq(ref="0")
 Drug_freq (ref="0")     Fluid_freq (ref="0")  HIV_freq(ref="0")  HPTN_C_freq(ref="0")  HPTN_UC_freq(ref="0")
 Hptothy_freq (ref="0")  LD_freq (ref="0")     Lymp_freq (ref="0")   METS_freq(ref="0")  Obesity_freq(ref="0")
 OthND_freq  (ref="0")    PCD_freq (ref="0")    PUD_NB_freq (ref="0")    PVD_freq(ref="0")   Para_freq(ref="0")
 Psycho_freq (ref="0")  RF_freq(ref="0")    WL_freq(ref="0")
 VD_freq(ref="0")  Tumor_freq(ref="0") Rheum_A_freq(ref="0")
 CBSA_URBN_RRL_IND (ref="R") gnrl_cd  (ref="PROFIT")
   BED_g  (ref="1")  MDCL_SCHL_AFLTN_CD (ref="1")
  five_score (ref="1");
 model death30(event="1")=los  y_treat age_g BENE_SEX_IDENT_CD RACE_CD_u
 medicaid hsh_g  admit_from icd_g
 Alcohol_freq   Arrhy_freq    BLA_freq          CHF_freq   COPD_freq
 Coag_freq      DA_freq      Dep_freq  Diab_C_freq  Diab_UC_freq
 Drug_freq      Fluid_freq   HIV_freq  HPTN_C_freq  HPTN_UC_freq
 Hptothy_freq   LD_freq      Lymp_freq    METS_freq  Obesity_freq
 OthND_freq      PCD_freq     PUD_NB_freq     PVD_freq   Para_freq
 Psycho_freq   RF_freq    WL_freq   VD_freq  Tumor_freq Rheum_A_freq
      CBSA_URBN_RRL_IND gnrl_cd
   BED_g    MDCL_SCHL_AFLTN_CD
   five_score
       /dist=binary solution link=logit oddsratio  DDFM=BW;
      random intercept/ subject=county_hos  type=vc;
 random intercept/ subject=prvdr_num (county_hos)  type=vc;

          run;
```

For the interaction term analysis in Table 3, we added one interaction term at a time into the main model. For example, for the interaction term of Medicaid and time in Table 3, the SAS command is below.

© 2022 Dang A et al. *JAMA Network Open.*

```
proc glimmix data=Bir_ana;
 class  county_HOS prvdr_num y_treat (ref="1") severity_g1(ref="1")
 age_g(ref="1") BENE_SEX_IDENT_CD (ref="1") RACE_CD_u(ref="1")
 medicaid(ref="0") hsh_g(ref="1")  admit_from (ref='community')
 icd_g (ref="g1_abd")
 Alcohol_freq (ref="0")  Arrhy_freq  (ref="0")  BLA_freq(ref="0")
 CHF_freq(ref="0")   COPD_freq(ref="0")
 Coag_freq(ref="0")       DA_freq (ref="0")     Dep_freq(ref="0")  Diab_C_freq(ref="0")  Diab_UC_freq(ref="0")
 Drug_freq (ref="0")      Fluid_freq (ref="0")  HIV_freq(ref="0")  HPTN_C_freq(ref="0")  HPTN_UC_freq(ref="0")
 Hptothy_freq (ref="0")  LD_freq (ref="0")     Lymp_freq (ref="0")   METS_freq(ref="0")  Obesity_freq(ref="0")
 OthND_freq  (ref="0")    PCD_freq (ref="0")    PUD_NB_freq (ref="0")    PVD_freq(ref="0")   Para_freq(ref="0")
 Psycho_freq (ref="0")  RF_freq(ref="0")    WL_freq(ref="0")
 VD_freq(ref="0")  Tumor_freq(ref="0") Rheum_A_freq(ref="0")
 CBSA_URBN_RRL_IND (ref="R") gnrl_cd  (ref="PROFIT")
   BED_g  (ref="1")  MDCL_SCHL_AFLTN_CD (ref="1")
  five_score (ref="1");
 model death30(event="1")=los  y_treat age_g BENE_SEX_IDENT_CD RACE_CD_u
 medicaid hsh_g  admit_from icd_g
 Alcohol_freq   Arrhy_freq    BLA_freq          CHF_freq   COPD_freq
 Coag_freq      DA_freq      Dep_freq  Diab_C_freq  Diab_UC_freq
 Drug_freq      Fluid_freq   HIV_freq  HPTN_C_freq  HPTN_UC_freq
 Hptothy_freq   LD_freq      Lymp_freq    METS_freq  Obesity_freq
 OthND_freq      PCD_freq     PUD_NB_freq     PVD_freq   Para_freq
 Psycho_freq   RF_freq    WL_freq   VD_freq  Tumor_freq Rheum_A_freq
      CBSA_URBN_RRL_IND gnrl_cd
   BED_g    MDCL_SCHL_AFLTN_CD
   five_score
   y_treat*medicaid
       /dist=binary solution link=logit oddsratio  DDFM=BW;
      random intercept/ subject=county_hos  type=vc;
 random intercept/ subject=prvdr_num (county_hos)  type=vc;

         run;
```

We checked the F statistics using type 3 tests of fixed effects for the interaction term. If the p-value was <0.05, we considered the interaction term to be significant. The image representing this is below.

© 2022 Dang A et al. *JAMA Network Open.*

Diab_C_freq	1	2405	277.16	<.0001
Diab_UC_freq	1	2393	0.07	0.7930
Drug_freq	1	2051	973.36	<.0001
Fluid_freq	1	2431	38798.2	<.0001
HIV_freq	1	906	0.23	0.6281
HPTN_C_freq	1	2398	1702.32	<.0001
HPTN_UC_freq	1	2430	4735.28	<.0001
Hptothy_freq	1	2400	1144.66	<.0001
LD_freq	1	2229	21169.2	<.0001
Lymp_freq	1	1900	1747.23	<.0001
METS_freq	1	2151	45458.8	<.0001
Obesity_freq	1	2328	4042.76	<.0001
OthND_freq	1	2365	56242.0	<.0001
PCD_freq	1	2189	2807.67	<.0001
PUD_NB_freq	1	1803	247.30	<.0001
PVD_freq	1	2297	1181.84	<.0001
Para_freq	1	2130	7380.95	<.0001
Psycho_freq	1	2069	39.42	<.0001
RF_freq	1	2415	2532.12	<.0001
WL_freq	1	2292	55070.0	<.0001
VD_freq	1	2234	316.11	<.0001
Tumor_freq	1	2302	8380.34	<.0001
Rheum_A_freq	1	2260	61.07	<.0001
CBSA_URBN_RRL_IND	1	2486	131.09	<.0001
GNRL_CD	2	500	47.81	<.0001
bed_g	3	610	5.65	0.0008
MDCL_SCHL_AFLTN_CD	3	532	5.66	0.0008
five_score	5	877	32.20	<.0001
y_treat*medicaid	1	2416	102.15	<.0001

Since the F statistic was significant (p<0.001), we further performed stratified analyses. The stratified model was the same as the main model in Table 1; however, we performed two models for the two sub-cohorts (one for Medicaid cohort and the other for the non-Medicaid cohort). Medicaid was not in the model because it was used to stratify the cohort. We repeated the models to check for the interaction term between time and other patient characteristics, or for the cross-level interaction term between time and hospital characteristics one-by-one. If the

© 2022 Dang A et al. *JAMA Network Open.*

F statistic was significant, we performed stratified analysis for the sub-cohorts by each characteristics.

The models for Table 2 are the same as the main model in Table 1. The only difference was the cohort. Table 1 used the cohort of year 2019 and April 2020 to March 2021. Table 2 used 6 quarterly cohorts: Q2 2020 vs. Q2 2019; Q3 2020 vs. Q3 2019; Q4 2020 vs. Q4 2019; Q1 2021 vs. Q1 2019; Q2 2021 vs. Q2 2019; and Q3 2021 vs. Q3 2019. Since the model was the same, the SAS code is not included here again.

Table 4 used the same 6 quarterly cohorts as in Table 2. We further stratified each quarterly cohort by the hospital SARS-CoV-2 prevalence in that pandemic quarter. We then performed the same model as that used in Table 1 for the sub-cohorts with high prevalence and low prevalence (12 models total). The odds ratios between 2020/2021 and 2019 were obtained for each sub-cohort. Since the model was the same, the SAS code is not included here again.

Table e1 used the same model as Table 1. The difference was the outcome was hospital mortality. Tables e6-e9 included the stratified analysis for 6 pandemic cohorts (2020 Q2-Q4, 2021 Q1-Q3). We performed the same model as in Table 1 for each sub-cohort stratified by each covariate. The models included SARS-CoV-2 prevalence and all other patient and hospital characteristics, except for the time and stratification variable.

All analyses were performed with SAS Enterprise version 7.1 (SAS Institute, Cary, NC) at the CMS Virtual Research Data Center.

© 2022 Dang A et al. *JAMA Network Open.*

eFigure 1. Cohort selection.

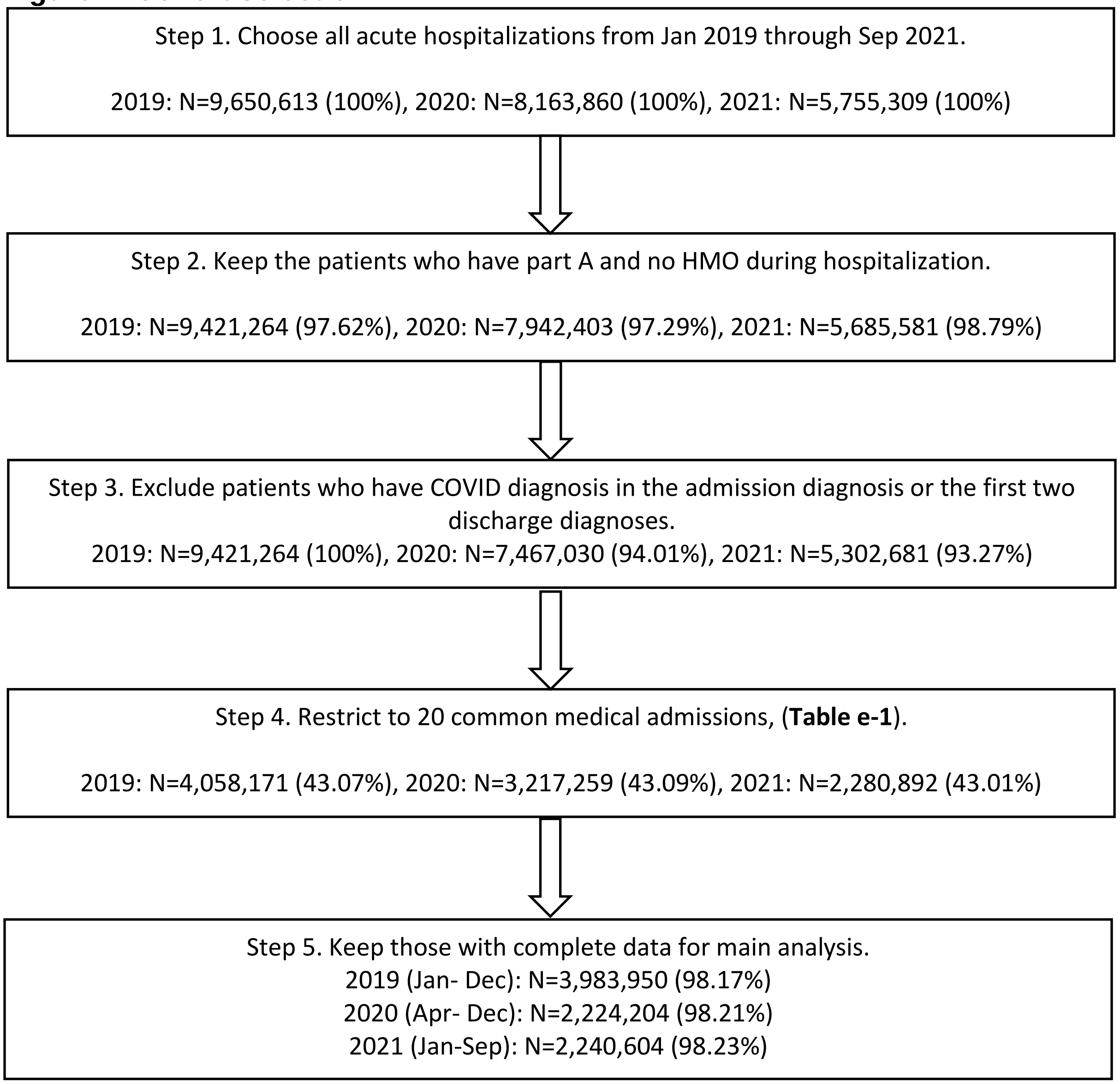

© 2022 Dang A et al. *JAMA Network Open.*

eFigure 2. Mortality rate in the 30 days after hospital admission from 12/1/19 to 9/30/21 for a non-SARS-CoV-2 medical diagnosis, stratified by census division.

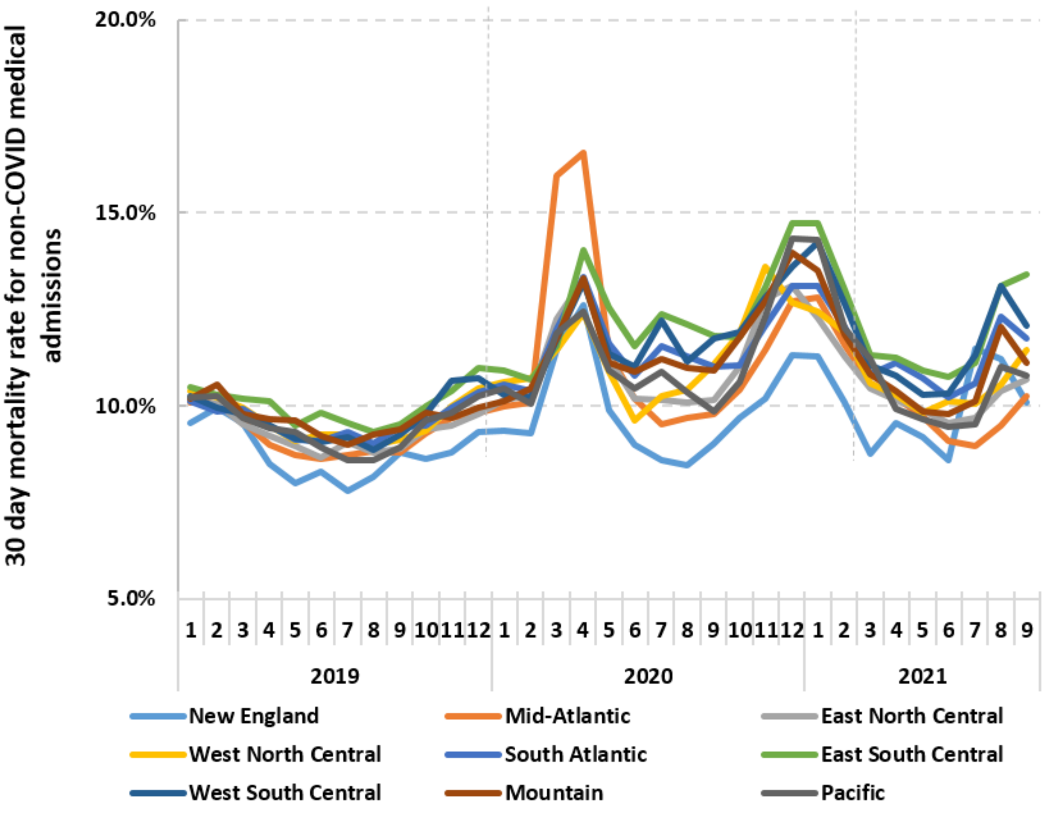

© 2022 Dang A et al. *JAMA Network Open.*

eFigure 3. Number of hospital admissions and their mortality in the 30 days after admission, for 20 common medical diagnoses. The analysis are similar to those in **Figure 1**, except stratified by each admission diagnosis. There are peaks in mortality around April 2020, January 2021 and September 2021 that are apparent for all diagnoses except for hip fracture and panceatitis, which did not have the September 2021 increase.

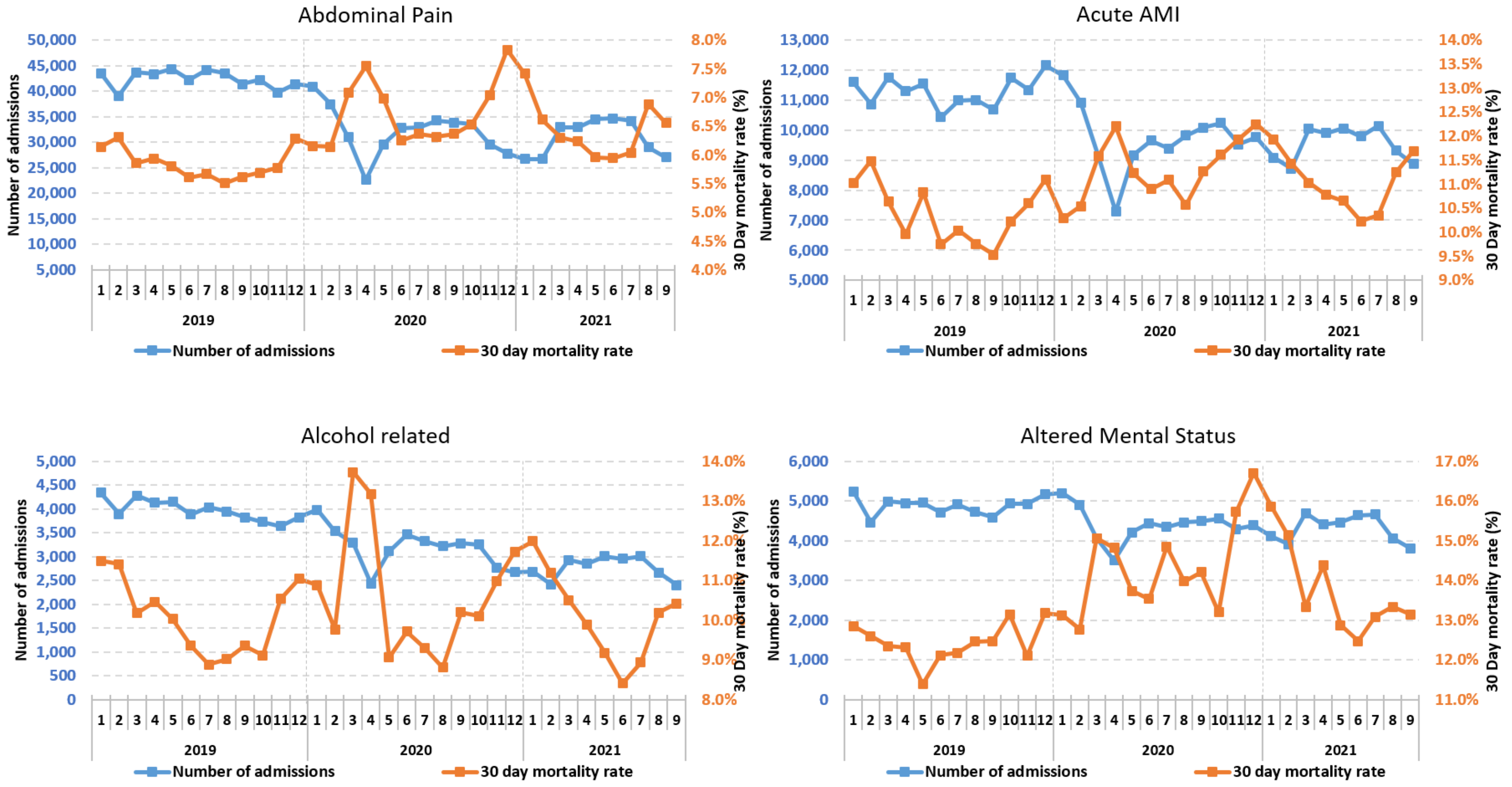

© 2022 Dang A et al. *JAMA Network Open.*

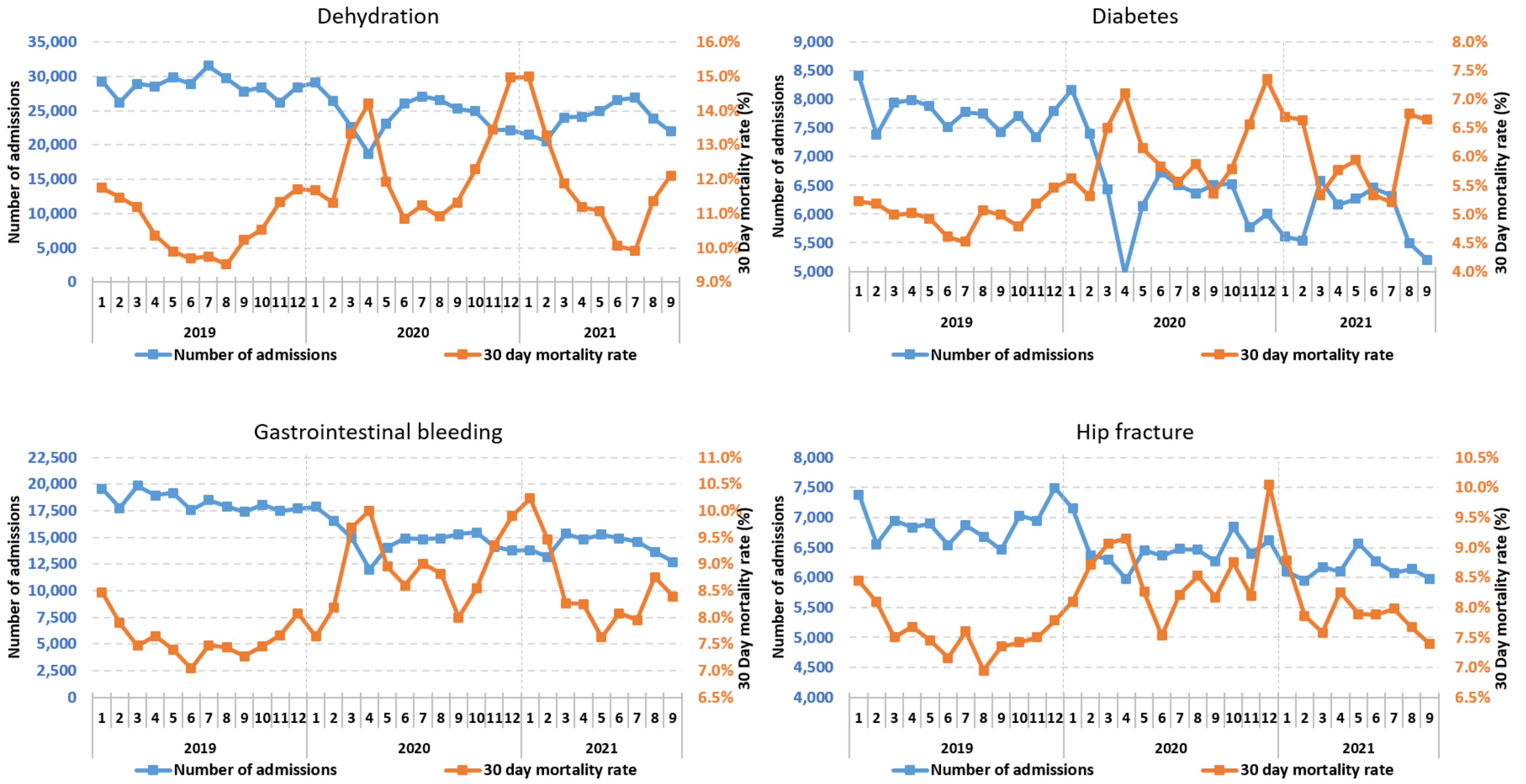

© 2022 Dang A et al. *JAMA Network Open.*

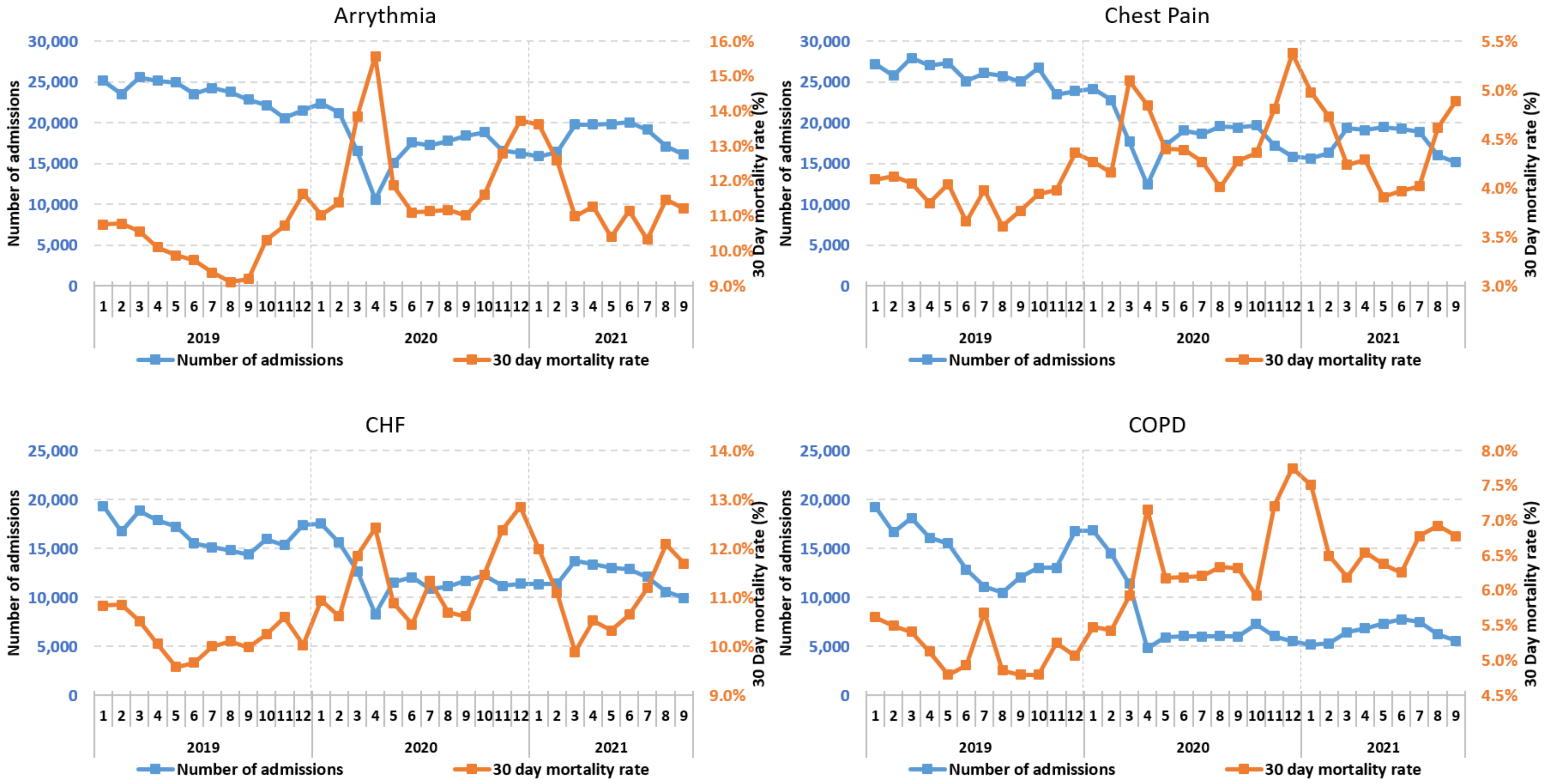

© 2022 Dang A et al. *JAMA Network Open.*

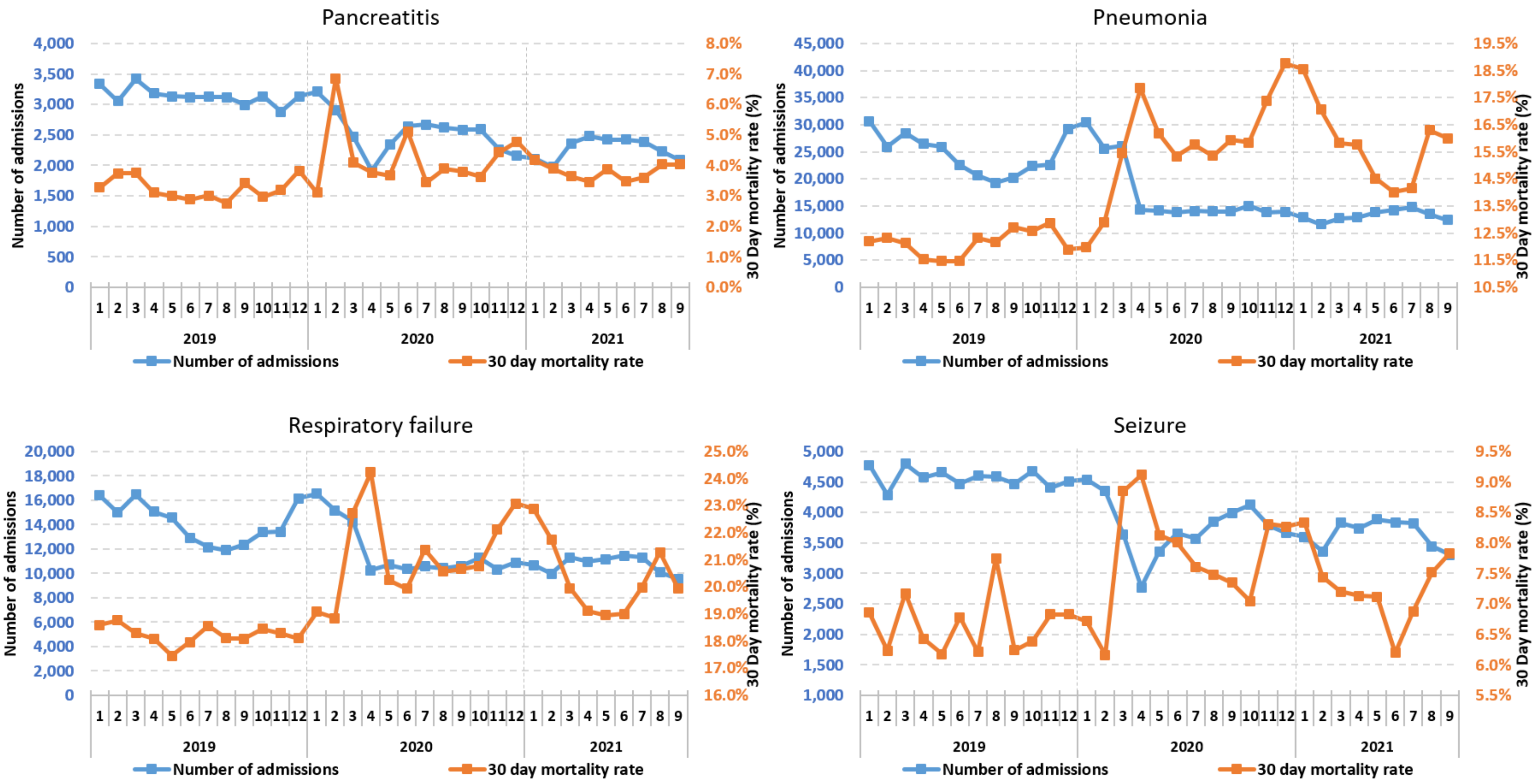

© 2022 Dang A et al. *JAMA Network Open.*

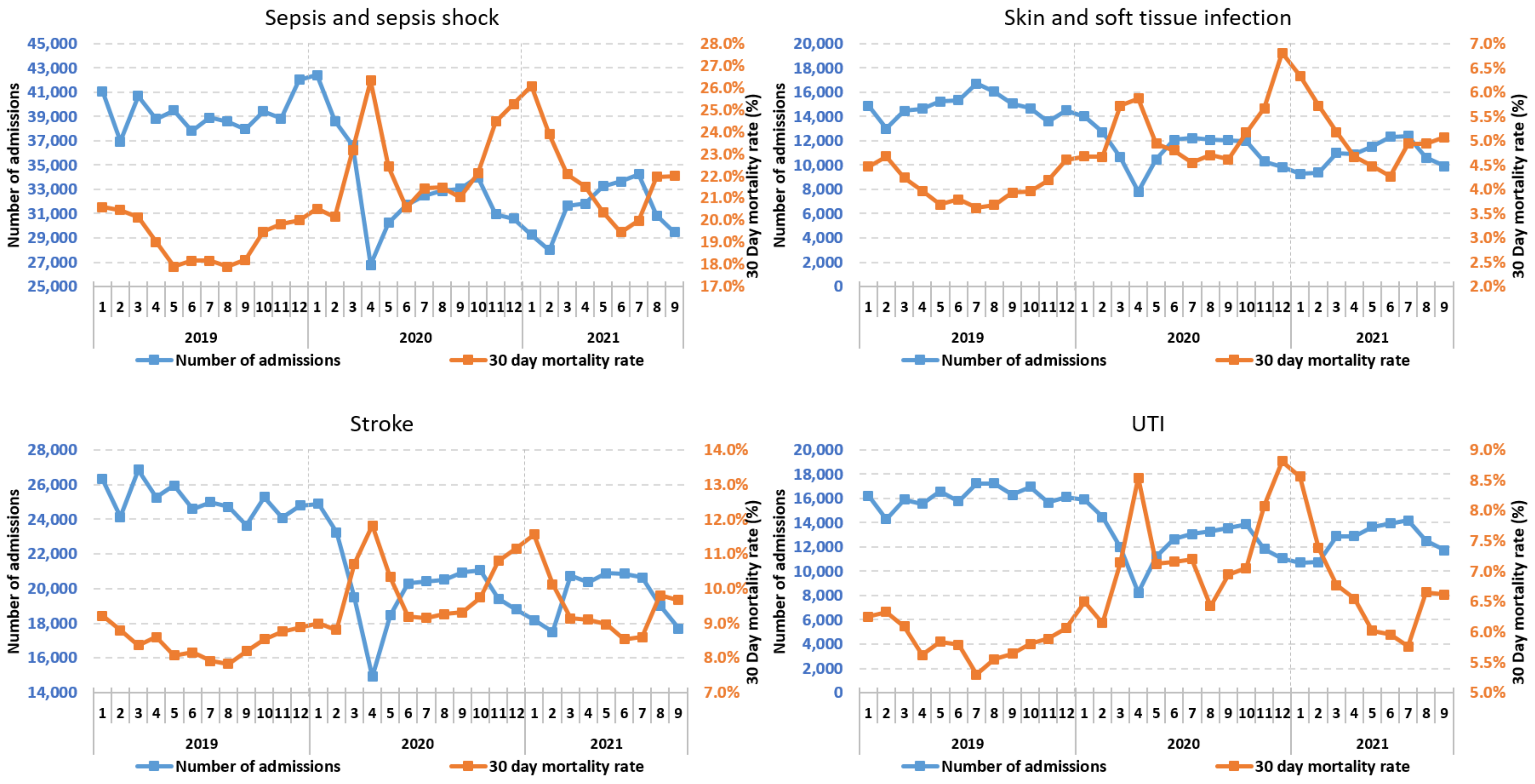

© 2022 Dang A et al. *JAMA Network Open.*

eTable 1. Unadjusted and adjusted 30-day mortality rates from a 2018 cohort for each of then 20 common admission diagnoses.[a]

Admission category	N (% 30-day mortality)	Adjusted rate (95% CI)
**Low severity diagnoses**		
Pancreatitis	7,661 (3.33%)	3.53% (3.12%, 3.99%)
UTI	38,093 (5.68%)	3.59% (3.44%, 3.76%)
Chest Pain	63,732 (3.82%)	3.97% (3.81%, 8.58%)
Skin and soft tissue infection	35,394 (3.98%)	4.06% (3.85%, 4.28%)
Diabetes	17,902 (4.67%)	4.54% (4.23%, 4.86%)
**Medium severity diagnoses**		
Abdominal pain	100,645 (5.76%)	4.71% (4.59%, 4.84%)
Seizure	11,016 (5.89%)	4.89% (4.51%, 5.29%)
COPD	36,670 (5.08%)	5.40% (5.16%, 5.65%)
Stroke	60,481 (8.43%)	5.67% (5.49%, 5.85%)
Gastrointestinal bleeding	44,453 (7.84%)	5.68% (5.48%, 5.88%)
Hip fracture	15,827 (7.45%)	5.91% (5.58%, 6.26%)
Dehydration	67,735 (10.53%)	5.93% (5.78%, 6.09%)
CHF	40,432 (10.30%)	6.13% (5.91%, 6.35%)
Altered Mental status	10,923 (12.58%)	7.15% (6.75%, 7.56%)
Arrythmia	54,910 (10.27%)	8.35% (8.13%, 8.58%)
**High severity diagnoses**		
Pneumonia	60,443 (12.23%)	8.40% (8.19%, 8.60%)
AMI	24,921 (11.03%)	9.83% (9.46%, 10.21%)
Alcohol-related	9,363 (11.26%)	11.01% (10.33%, 11.70%)
Sepsis and sepsis shock	87,438 (19.68%)	11.98% (11.77%, 12.19%)
Respiratory failure	31,804 (18.72%)	13.78% (13.41%, 14.15%)

[a]For analyses exploring interactions between admission characteristics and hospital prevalence of SARS-CoV-2, (Tables e5-e10), we substituted a “disease severity” measure for the 20 individual admission diagnoses. In order to generate a measure of severity for the 20 admission diagnoses, we calculated the unadjusted and adjusted mortality in the 30 days post admission using 2018 fee for service Medicare data. In the adjusted analyses we controlled for all the admission characteristics used in the analyses in **Table 1** including the comorbidities listed in **Table e3**. Thus, the mortality rate associated with each diagnosis is independent of other admission characteristics.

CI: confidence interval; UTI: urinary tract infection; COPD: Chronic obstructive pulmonary disease; CHF: congestive heart failure; AMI: acute myocardial Infarction.

eTable 2. Characteristics of non-COVID admissions in 2019 and April 2020-September 2021, along with 30-day mortality rates.

Characteristic	Jan 2019 - Dec2019	30 day mortality<br><br>N (%)	Apr 2020 - Sep 2021	30 day mortality<br><br>N (%)
All	3,983,950 (100%)	375,605 (9.43%)	4,464,808 (100%)	496,229 (11.11%)
Age (Per year)				
<=65	858,813 (21.56%)	45,494 (5.30%)	906,561 (20.30%)	58,944 (6.50%)
66-70	624,961 (15.69%)	44,298 (7.09%)	716,771 (16.05%)	60,582 (8.45%)
71-75	636,165 (15.97%)	52,213 (8.21%)	746,015 (16.71%)	73,319 (9.83%)
75-80	602,157 (15.11%)	57,239 (9.51%)	698,648 (15.65%)	79,218 (11.34%)
81-85	533,399 (13.39%)	60,577 (11.36%)	608,846 (13.64%)	80,724 (13.26%)
86+	728,455 (18.28%)	115,784 (15.89%)	787,967 (17.65%)	143,442 (18.20%)
Gender				
Male	1,869,002 (46.91%)	186,199 (9.96%)	2,117,523 (47.43%)	247,986 (11.71%)
Female	2,114,948 (53.09%)	189,406 (8.96%)	2,347,285 (52.57%)	248,243 (10.58%)
Race				
White	3,077,794 (77.25%)	299,064 (9.72%)	3,468,026 (77.67%)	391,035 (11.28%)
Black	476,762 (11.97%)	39,479 (8.28%)	510,393 (11.43%)	510,393 (11.43%)
Hispanic	256,040 (6.43%)	256,040 (8.42%)	278,330 (6.23%)	29,448 (10.58%)
Other	173,354 (4.35%)	15,500 (8.94%)	208,059 (4.66%)	22,672 (10.90%)
Medicaid				
No	2,833,520 (71.12%)	279,451 (9.86%)	3,273,820 (73.32%)	373,172 (11.40%)
Yes	1,150,430 (28.88%)	96,154 (8.36%)	1,190,988 (26.68%)	123,057 (10.33%)
Education (Percent of persons age 25+ in Zip area with high school education) (Per percent)				
Q1	1,048,242 (26.31%)	97,944 (9.34%)	1,112,379 (24.91%)	127,550 (11.47%)
Q2	1,016,381 (25.51%)	96,621 (9.51%)	1,126,069 (25.22%)	127,091 (11.29%)
Q3	1,001,517 (25.14%)	94,964 (9.48%)	1,144,198 (25.63%)	125,730 (10.99%)
Q4	917,810 (23.04%)	86,076 (9.38%)	1,082,162 (24.24%)	115,858 (10.71%)
Residence prior to hospitalization				
Community	3,502,381 (87.91%)	302,149 (8.63%)	3,890,813 (87.14%)	398,486 (10.24%)
Nursing facility or other institutions	481,569 (12.09%)	73,456 (15.25%)	573,995 (12.86%)	97,743 (17.03%)
Admission category				
g1-Abdominal pain	489,870 (12.30%)	28,211 (5.76%)	541,484 (12.13%)	35,283 (6.52%)
g2-AMI	129,581 (3.25%)	13,448 (10.38%)	165,834 (3.71%)	18,518 (11.17%)
g3-Alcohol-related	45,575 (1.14%)	4,545 (9.97%)	50,905 (1.14%)	5,111 (10.04%)
g4-Altered Mental status	55,781 (1.40%)	6,905 (12.38%)	74,845 (1.68%)	10,480 (14.00%)
g5-Arrythmia	272,393 (6.84%)	27,557 (10.12%)	302,466 (6.77%)	35,030 (11.58%)
g6-Chest Pain	300,849 (7.55%)	11,742 (3.90%)	310,078 (6.94%)	13,652 (4.40%)
g7-CHF	190,264 (4.78%)	19,362 (10.18%)	203,195 (4.55%)	22,670 (11.16%)
g8-COPD	167,997 (4.22%)	8,641 (5.14%)	109,569 (2.45%)	7,151 (6.53%)
g9-Dehydration	329,931 (8.28%)	34,622 (10.49%)	418,391 (9.37%)	49,522 (11.84%)
g10-Diabetes	89,028 (2.23%)	4,393 (4.93%)	105,940 (2.37%)	6,361 (6.00%)
g11-	211,469 (5.31%)	15,904 (7.52%)	250,275 (5.61%)	21,763 (8.70%)

© 2022 Dang A et al. *JAMA Network Open.*

Gastrointestinal bleeding				
g12-Hip fracture	78,713 (1.98%)	5,826 (7.40%)	109,316 (2.45%)	8,839 (8.09%)
g13-Pancreatitis	36,201 (0.91%)	1,146 (3.17%)	41,107 (0.92%)	1,610 (3.92%)
g14-Pneumonia	280,393 (7.04%)	33,669 (12.01%)	238,472 (5.34%)	38,248 (16.04%)
g15-Respiratory failure	160,736 (4.03%)	29,150 (18.14%)	185,821 (4.16%)	38,550 (20.75%)
g16-Seizure	52,289 (1.31%)	3,356 (6.42%)	63,469 (1.42%)	4,772 (7.52%)
g17- Sepsis and sepsis shock	446,417 (11.21%)	85,161 (19.08%)	546,215 (12.23%)	121,082 (22.17%)
g18-Skin and soft tissue infection	170,649 (4.28%)	6,803 (3.99%)	190,525 (4.27%)	9,626 (5.05%)
g19-stroke	289,497 (7.27%)	24,351 (8.41%)	341,290 (7.64%)	32,969 (9.66%)
g20-UTI	186,317 (4.68%)	10,813 (5.80%)	215,611 (4.83%)	14,992 (6.95%)
Length of stay, Median (Q1, Q3)	5 (3, 7)	-	5 (3, 7)	-
Elixhauser Score Median (Q1, Q3)	4 (3, 6)	-	4 (3, 6)	-

AMI: acute myocardial Infarction; CHF: congestive heart failure; COPD: Chronic obstructive pulmonary disease; UTI: urinary tract infection; STD: standard deviation.

Given the very large sample size, all differences in the characteristics in 4/1/20-3/3121 were statistically significant compared to those in 2019 at P < 0.05 level. Also, all differences in mortality rates between categories of characteristics were statistically significant at P<0.05.

© 2022 Dang A et al. *JAMA Network Open.*

eTable 3. Comorbidities included in the multilevel logistic regression model presented in Table 1 for 30 day mortality after admission, for non-SARS-CoV-2 medical admissions during January to December 2019 and April 2020 to March 2021, unadjusted rates and odds from three-level (admission, hospital, county) logistic regression analysis, comparing mortality in April 2020 to March 2021 to mortality in 2019.

.

**Elixhauser comorbidity**	N (%)	30-day mortality after admission<br><br>N (%)	Odds Ratio (95% confidence interval)
All	6,949,196	712,708 (10.30%)	
Alcohol abuse			
No	6,640,112 (95.97%)	687,706 (10.36%)	Reference
Yes	279,084 (4.03%)	25,002 (8.96%)	0.86 (0.85-0.88)
Cardiac Arrhythmia			
No	4,639,899 (67.06%)	404,515 (8.72%)	Reference
Yes	2,279,297 (32.94%)	308,193 (13.52%)	1.25 (1.24-1.26)
Blood Loss Anemia			
No	6,849,839 (99.00%)	705,003 (10.29%)	Reference
Yes	69,357 (1.00%)	7,705 (11.11%)	0.96 (0.93-0.98)
Congestive Heart Failure			
No	4,597,810 (66.45%)	393,520 (8.56%)	Reference
Yes	1,391,525 (33.55%)	319,188 (13.75%)	1.45 (1.44-1.46)
Chronic Obstructive Pulmonary Disease			
No	4,974,958 (71.90%)	497,017 (9.99%)	Reference
Yes	1,944,238 (28.10%)	215,691 (11.09%)	1.06 (1.05-1.06)
Coagulopathy			
No	6,409,331 (92.63%)	617,902 (9.64%)	Reference
Yes	509,865 (7.37%)	94,806 (18.59%)	1.57 (1.56-1.58)
Deficiency Anemia			
No	6,519,998 (94.23%)	670,156 (10.28%)	Reference
Yes	399,198 (5.77%)	42,552 (10.66%)	0.83 (0.82-0.84)
Depression			
No	5,851,878 (84.57%)	626,668 (10.71%)	Reference
Yes	1,067,318 (15.49%)	86,040 (8.06%)	0.84 (0.83-0.84)
Diabetes Complicated			
No	5,079,587 (73.41%)	518,093 (10.20%)	Reference
Yes	1,839,609 (26.59%)	194,615 (10.58%)	1.06 (1.05-1.07)
Diabetes Uncomplicated			
No	6,090,793 (88.03%)	640,919 (10.52%)	Reference
Yes	828,403 (11.97%)	71,789 (8.67%)	1.00 (0.99-1.01)
Drug Abuse			
No	6,688,065 (96.66%)	700,378 (10.47%)	Reference
Yes	231,131 (3.34%)	12,330 (5.33%)	0.73 (0.72-0.74)
Fluid and Electrolyte Disorders			
No	4,095,997 (59.20%)	295,668 (7.22%)	Reference
Yes	2,823,199 (40.80%)	417,040 (14.77%)	1.77 (1.76-1.78)
AIDS/HIV			
No	6,903,438 (99.77%)	711,413 (10.31%)	Reference
Yes	15,758 (0.23%)	1,295 (8.22%)	1.02 (0.95-1.08)
Hypertension complicated			

© 2022 Dang A et al. *JAMA Network Open.*

No	4,248,489 (61.40%)	381,647 (8.98%)	Reference
Yes	2,670,707 (38.60%)	331,061 (12.40%)	0.85 (0.84-0.86)
Hypertension Uncomplicated			
No	4,429,887 (64.02%)	531,257 (11.99%)	Reference
Yes	2,489,309 (35.98%)	181,451 (7.29%)	0.76 (0.75-0.77)
Hypothyroidism			
No	5,529,087 (79.91%)	572,083 (10.35%)	Reference
Yes	1,390,109 (20.09%)	140,625 (10.12%)	0.89 (0.88-0.90)
Liver Disease			
No	6,506,951 (94.04%)	641,752 (9.86%)	Reference
Yes	412,245 (5.96%)	70,956 (17.21%)	2.12 (2.10-2.14)
Lymphoma			
No	6,832,315 (98.74%)	698,225 (10.22%)	Reference
Yes	86,881 (1.26%)	14,483 (16.67%)	1.52 (1.49-1.55)
Metastatic Cancer			
No	6,680,539 (96.55%)	634,783 (9.50%)	Reference
Yes	238,657 (3.45%)	77,925 (32.65%)	3.77 (3.72-3.81)
Obesity			
No	5,789,202 (83.67%)	639,263 (11.04%)	Reference
Yes	1,129,994 (16.33%)	73,445 (6.50%)	0.75 (0.75-0.75)
Other Neurological Disorders			
No	5,729,974 (82.81%)	488,110 (8.52%)	Reference
Yes	1,189,222 (17.19%)	224,598 (18.89%)	2.14 (2.12-2.15)
Pulmonary Circulation Disorders			
No	6,419,732 (92.78%)	635,622 (9.90%)	Reference
Yes	499,464 (7.22%)	77,086 (15.43%)	1.29 (1.27-1.30)
Peptic Ulcer Disease excluding bleeding			
No	6,850,158 (99.00%)	707,388 (10.33%)	Reference
Yes	69,038 (1.00%)	5,320 (7.71%)	0.79 (0.76-0.81)
Peripheral Vascular Disorders			
No	6,394,884 (92.42%)	647,219 (10.12%)	Reference
Yes	524,312 (7.58%)	65,489 (12.49%)	1.18 (1.17-1.19)
Paralysis			
No	6,714,487 (97.04%)	682,302 (10.16%)	Reference
Yes	204,709 (2.96%)	30,406 (14.85%)	1.90 (1.87-1.93)
Psychoses			
No	6,780,423 (97.99%)	701,664 (10.35%)	Reference
Yes	138,773 (2.01%)	11,044 (7.96%)	0.94 (0.92-0.96)
Renal Failure			
No	4,711,961 (68.10%)	434,998 (9.23%)	Reference
Yes	2,207,235 (31.90%)	277,710 (12.58%)	1.21 (1.20-1.22)
Weight Loss			
No	6,210,494 (89.76%)	547,279 (8.81%)	Reference
Yes	708,702 (10.24%)	165,429 (23.34%)	2.37 (2.35-2.39)
Valvular Disease			
No	6,244,684 (90.25%)	630,728 (10.10%)	Reference
Yes	674,512 (9.75%)	81,980 (12.15%)	0.92 (0.92-0.93)
Solid Tumor without Metastasis			
No	6,512,210 (94.12%)	617,223 (9.48%)	Reference
Yes	406,986 (5.88%)	95,485 (23.46%)	1.64 (1.62-1.65)
Rheumatoid Arthritis/collagen			
No	6,608,449 (95.51%)	685,381 (10.37%)	Reference

© 2022 Dang A et al. *JAMA Network Open.*

Yes	310,747 (4.49%)	27,327 (8.79%)	0.95 (0.93-0.96)

OR: odds ratio; CI: confidence interval.

eTable 4. Hospital mortality for non-SARS-CoV-2 medical admissions during January to December 2019 and April 2020 to March 2021, unadjusted rates and odds from three-level (admission, hospital, county) logistic regression analysis.[a]

Characteristic	Admissions (%)	Hospital mortality N (%)	Odds Ratio (95% Confidence Interval)
All	6,919,196	301,962 (4.36%)	
Year			
Jan 2019- Dec 2019	3,983,950 (57.58%)	158,076 (3.97%)	Reference
Apr 2020- Mar 2021	2,935,246 (42.42%)	143,886 (4.90%)	1.16 (1.15-1.17)
Length of Stay		-	1.002 (1.001-1.002)
Age (Per year)			
<=65	1,472,200 (21.28%)	44,549 (3.03%)	Reference
66-70	1,096,723 (15.85%)	41,503 (3.78%)	1.21 (1.19-1.23)
71-75	1,121,875 (16.21%)	46,897 (4.18%)	1.34 (1.32-1.36)
75-80	1,055,721 (15.26%)	48,092 (4.56%)	1.46 (1.44-1.48)
81-85	930,264 (13.44%)	46,097 (4.96%)	1.61 (1.59-1.63)
86+	1,242,413 (17.96%)	74,824 (6.02%)	2.05 (2.02-2.08)
Gender			
Male	3,266,768 (47.21%)	154,499 (4.73%)	Reference
Female	3,652,428 (52.79%)	147,463 (4.04%)	0.97 (0.96-0.97)
Race			
White	5,351,956 (77.35%)	229,928 (4.30%)	Reference
Black	821,569 (11.87%)	36,609 (4.46%)	0.99 (0.97-1.01)
Hispanic	438,453 (6.34%)	20,185 (4.60%)	1.05 (1.03-1.07)
Other[b]	307,218 (4.44%)	15,240 (4.96%)	1.07 (1.05-1.09)
Medicaid			
No	4,973,602 (71.88%)	221,573 (4.45%)	Reference
Yes	1,945,594 (28.12%)	80,389 (4.13%)	0.92 (0.91-0.93)
Education (Percent of persons age 25+ in Zip area with high school education) (Per percent)			
Q1	1,788,498 (25.85%)	82,834 (4.63%)	Reference
Q2	1,761,020 (25.45%)	77,476 (4.40%)	0.98 (0.97-0.99)
Q3	1,750,825 (25.30%)	74,737 (4.27%)	0.95 (0.94-0.96)
Q4	1,618,853 (23.40%)	66,915 (4.13%)	0.92 (0.91-0.93)
Residence prior to hospitalization			
Community	6,060,688 (87.59%)	233,056 (3.85%)	Reference
Nursing facility or other institutions	858,508 (12.41%)	68,906 (8.03%)	1.76 (1.74-1.77)
Admission category			
Abdominal pain	843,966 (12.20%)	18,259 (2.16%)	Reference
AMI	239,076 (3.46%)	14,468 (6.05%)	2.94 (2.88-3.01)
Alcohol-related	80,080 (1.16%)	4,313 (5.39%)	2.43 (2.35-2.52)
Altered Mental status	105,459 (1.52%)	3,964 (3.76%)	1.10 (1.06-1.14)
Arrythmia	466,700 (6.75%)	33,079 (7.09%)	3.26 (3.19-3.32)
Chest Pain	505,765 (7.31%)	8,109 (1.60%)	0.91 (0.89-0.94)
CHF	323,536 (4.68%)	11,131 (3.44%)	1.13 (1.10-1.16)
COPD	237,393 (3.43%)	3,629 (1.53%)	0.92 (0.89-0.95)
Dehydration	604,055 (8.73%)	19,363 (3.21%)	0.97 (0.95-0.99)

Diabetes	160,147 (2.31%)	2,816 (1.76%)	0.82 (0.79-0.85)
Gastrointestinal bleeding	378,139 (5.47%)	11,058 (2.92%)	1.14 (1.11-1.17)
Hip fracture	152,156 (2.20%)	3,342 (2.20%)	1.09 (1.05-1.13)
Pancreatitis	63,682 (3.53%)	941 (1.48%)	0.83 (0.78-0.79)
Pneumonia	439,673 (6.35%)	21,246 (4.83%)	1.91 (1.87-1.95)
Respiratory failure	284,115 (4.11%)	28,683 (10.10%)	3.94 (3.86-4.02)
Seizure	94,404 (1.36%)	2,107 (2.23%)	0.84 (0.80-0.88)
Sepsis and sepsis shock	805,579 (11.64%)	88,313 (10.96%)	3.39 (3.33-3.45)
Skin and soft tissue infection	295,468 (4.27%)	4,578 (1.55%)	0.86 (0.83-0.89)
Stroke	514,512 (7.44%)	18,427 (3.58%)	1.37 (1.34-1.40)
UTI	325,291 (4.70%)	4,136 (1.27%)	0.48 (0.46-0.50)
Hospital or county characteristics			
Location			
Rural	907,417 (13.11%)	33,004 (3.64%)	Reference
Urban	6,011,779 (86.89%)	268,958 (4.47%)	0.94 (0.90-0.98)
Type of provider			
For profit	1,037,227 (14.99%)	42,566 (4.10%)	Reference
Government	854,820 (12.35%)	38,679 (4.52%)	1.13 (1.08-1.18)
Non-profit	5,027,149 (72.66%)	220,717 (4.39%)	0.99 (0.95-1.03)
Bed size			
<=200	1,820,950 (26.32%)	64,493 (3.54%)	Reference
201-350	1,835,572 (26.53%)	79,637 (4.34%)	1.19 (1.15-1.23)
351-500	1,226,921 (17.73%)	58,013 (4.73%)	1.28 (1.22-1.34)
>=501	2,035,753 (29.42%)	99,819 (4.90%)	1.28 (1.22-1.34)
Medical school affiliation			
Major	1,610,211 (23.27%)	79,160 (4.92%)	Reference
Limited	1,503,721 (21.73%)	67,579 (4.49%)	0.98 (0.94-1.03)
Graduate	345,069 (4.99%)	17,283 (5.01%)	1.04 (0.97-1.12)
No affiliation	3,460,195 (50.01%)	137,940 (3.99%)	0.97 (0.92-1.01)
HCAHPS Summary Star			
1	156,679 (2.26%)	8,411 (5.37%)	Reference
2	896,157 (12.95%)	41,614 (4.64%)	0.92 (0.85-0.99)
3	3,460,499 (50.01%)	152,760 (4.41%)	0.86 (0.79-0.92)
4	1,984,041 (28.67%)	83,748 (4.22%)	0.81 (0.75-0.88)
5	237,628 (3.43%)	8,943 (3.76%)	0.75 (0.68-0.83)
Not available	184,192 (2.66%)	6,486 (3.52%)	0.96 (0.88-1.04)

[a]The models also include 31 comorbidities shown in **Table e3.**

AMI: acute myocardial Infarction; CHF: congestive heart failure; COPD: Chronic obstructive pulmonary disease; UTI: urinary tract infection; HCAHPS: Hospital Consumer Assessment of Healthcare Providers and Systems.

© 2022 Dang A et al. *JAMA Network Open.*

eTable 5. Odds of mortality in the 30 days after hospital admission for patients with non-SARS-CoV-2 medical diagnoses from April through June of 2020 by quartile of prevalence of SARS-CoV-2 in the hospital, stratified by characteristics of the admission.[a]

Admission/hospital characteristic	SARS-CoV-2 prevalence 0	SARS-CoV-2 prevalence 0.01-2.05% OR (95% CI)	SARS-CoV-2 prevalence >2.06% OR (95% CI)
Disease severity			
Low	Ref	0.98 (0.90, 1.06)	0.98 (0.90, 1.07)
Medium	Ref	0.99 (0.95, 1.04)	1.01 (0.96, 1.07)
High	Ref	1.04 (0.99, 1.10)	1.21 (1.14, 1.28)
Age			
<=65	Ref	1.11 (1.03, 1.21)	1.20 (1.10, 1.31)
66-70	Ref	1.06 (0.98, 1.15)	1.11 (1.12, 1.21)
71-75	Ref	1.06 (0.99, 1.15)	1.10 (1.02, 1.20)
75-80	Ref	1.03 (0.95, 1.11)	1.14 (1.06, 1.23)
81-85	Ref	0.98 (0.91, 1.06)	1.03 (0.96, 1.11)
86+	Ref	0.92 (0.87, 0.98)	1.02 (0.96, 1.09)
Race			
White	Ref	1.01 (0.98, 1.05)	1.08 (1.03, 1.13)
Black	Ref	1.04 (0.91, 1.19)	1.12 (0.99, 1.28)
Hispanic	Ref	0.95 (0.84, 1.08)	0.98 (0.86, 1.12)
Other	Ref	1.01 (0.88, 1.16)	1.21 (1.05, 1.39)
Medicaid			
No	Ref	0.99 (0.95, 1.03)	1.07 (1.02, 1.12)
Yes	Ref	1.07 (1.01, 1.14)	1.14 (1.07, 1.22)
Residence prior to hospitalization			
Community	Ref	0.98 (0.94, 1.02)	1.05 (0.99, 1.09)
Nursing facility or other institutions	Ref	1.18 (1.09, 1.28)	1.35 (1.24, 1.47)
Medical school affiliation			
Major	Ref	0.91 (0.77, 1.07)	0.95 (0.81, 1.12)
Limited	Ref	1.04 (0.94, 1.15)	1.19 (1.07, 1.33)
Graduate	Ref	0.98 (0.63, 1.53)	0.97 (0.61, 1.55)
No affiliation	Ref	1.02 (0.97, 1.07)	1.09 (1.04, 1.15)
Location			
Rural	Ref	0.94 (0.88, 1.01)	1.14 (1.06, 1.23)
Urban	Ref	1.03 (0.98, 1.08)	1.09 (1.03, 1.15)
Type of hospital			
For profit	Ref	1.01 (0.91, 1.12)	1.08 (0.97, 1.20)
Government	Ref	0.97 (0.87, 1.09)	1.04 (0.93, 1.16)
Non-profit	Ref	1.02 (0.97, 1.07)	1.10 (1.04, 1.16)
Hospital quality score			
1	Ref	1.37 (0.88, 2.14)	1.30 (0.84, 2.02)
2	Ref	1.00 (0.89, 1.12)	1.08 (0.96, 1.22)
3	Ref	1.04 (0.98, 1.10)	1.13 (1.06, 1.20)
4	Ref	0.99 (0.92, 1.08)	1.04 (0.95, 1.14)
5	Ref	1.00 (0.79, 1.26)	1.12 (0.84, 1.49)

© 2022 Dang A et al. *JAMA Network Open.*

[a]In generating the data presented in Tables e5-e10, we first tested for interactions between admission or hospital characteristics and the percentages of SARS-CoV-2 admissions at each hospital (prevalence of SARS-CoV-2). Tables 5-10 present the stratified analyses based on those interactions. Each table presents analyses from a three-month period, because SARS-CoV-2 prevalence in hospitals changed over time. If an admission or hospital characteristic showed a significant interaction with hospital SARS-CoV-2 prevalence in **any time period**, then it was included in the stratified analyses for all the time periods. .
OR, odds ratio; CI, confidence interval.

© 2022 Dang A et al. *JAMA Network Open.*

eTable 6. Odds of mortality in the 30 days after hospital admission for patients with non-SARS-CoV-2 medical diagnoses from July through September of 2020 by level of prevalence of SARS-CoV-2 in the hospital, stratified by characteristics of the admission.[a]

**Admission/hospital characteristic**	SARS-CoV-2 prevalence 0 OR (95% CI)	SARS-CoV-2 prevalence 0.01-3.93% OR (95% CI)	SARS-CoV-2 prevalence >3.94% OR (95% CI)
Disease severity			
Low	Ref	0.90 (0.83, 0.99)	1.05 (0.95, 1.16)
Medium	Ref	0.97 (0.92, 1.03)	1.09 (1.03, 1.16)
High	Ref	1.06 (0.99, 1.13)	1.23 (1.15, 1.31)
Age			
<=65	Ref	1.17 (1.06, 1.29)	1.38 (1.24, 1.53)
66-70	Ref	1.00 (0.91, 1.10)	1.12 (1.01, 1.24)
71-75	Ref	1.04 (0.95, 1.14)	1.15 (1.05, 1.27)
75-80	Ref	1.01 (0.93, 1.10)	1.18 (1.07, 1.30)
81-85	Ref	0.95 (0.87, 1.03)	1.13 (1.04, 1.24)
86+	Ref	0.92 (0.86, 0.98)	1.03 (0.96, 1.10)
Race			
White	Ref	1.00 (0.95, 1.04)	1.12 (1.06, 1.18)
Black	Ref	1.07 (0.93, 1.22)	1.27 (1.10, 1.47)
Hispanic	Ref	0.87 (0.76, 1.01)	1.14 (1.98, 1.33)
Other	Ref	1.06 (0.90, 1.25)	1.14 (0.95, 1.37)
Medicaid			
No	Ref	0.97 (0.93, 1.02)	1.08 (1.02, 1.14)
Yes	Ref	1.06 (0.99, 1.13)	1.29 (1.19, 1.39)
Residence prior to hospitalization			
Community	Ref	0.97 (0.93, 1.02)	1.13 (1.07, 1.18)
Nursing facility or other institutions	Ref	1.13 (1.03, 1.23)	1.16 (1.05, 1.28)
Medical school affiliation			
Major	Ref	1.02 (0.89, 1.17)	1.30 (1.11, 1.52)
Limited	Ref	1.02 (0.93, 1.13)	1.15 (1.03, 1.29)
Graduate	Ref	0.67 (0.46, 0.97)	0.95 (0.64, 1.44)
No affiliation	Ref	0.99 (0.94, 1.04)	1.14 (1.08, 1.21)
Location			
Rural	Ref	0.95 (0.88, 1.02)	1.16 (1.08, 1.25)
Urban	Ref	1.01 (0.95, 1.06)	1.12 (1.05, 1.19)
Type of hospital			
For profit	Ref	1.10 (0.97, 1.24)	1.20 (1.06, 1.36)
Government	Ref	0.92 (0.82, 1.04)	1.09 (0.98, 1.23)
Non-profit	Ref	0.98 (0.93, 1.03)	1.14 (1.07, 1.21)
Hospital quality score			
1	Ref	0.97 (0.71, 1.33)	1.25 (0.81, 1.92)
2	Ref	1.16 (1.02, 1.31)	1.32 (1.15, 1.52)
3	Ref	1.02 (0.95, 1.09)	1.18 (1.09, 1.27)
4	Ref	0.96 (0.87, 1.06)	1.12 (1.01, 1.24)
5	Ref	1.04 (0.85, 1.27)	1.20 (0.94, 1.53)

[a]See footnote to Table e5.
OR, odds ratio; CI, confidence interval.

© 2022 Dang A et al. *JAMA Network Open.*

eTable 7. Odds of mortality in the 30 days after hospital admission for patients with non-SARS-CoV-2 medical conditions from October through December of 2020 by quartile of prevalence of SARS-CoV-2 in the hospital, stratified by characteristics of the admission.[a]

**Admission/hospital characteristic**	SARS-CoV-2 prevalence 0-2.87% OR (95% CI)	SARS-CoV-2 prevalence 2.88-7.09% OR (95% CI)	SARS-CoV-2 prevalence 7.10-14.96% OR (95% CI)	SARS-CoV-2 prevalence >14.97% OR (95% CI)
Disease severity				
Low	Ref	0.95 (0.89, 1.02)	1.01 (0.94, 1.08)	1.10 (1.01, 1.21)
Medium	Ref	1.05 (1.01, 1.09)	1.09 (1.04, 1.13)	1.20 (1.13, 1.27)
High	Ref	1.07 (1.02, 1.12)	1.12 (1.07, 1.17)	1.19 (1.13, 1.27)
Age (Per year)				
<=65	Ref	1.01 (0.95, 1.08)	1.05 (0.98, 1.13)	1.00 (0.91, 1.11)
66-70	Ref	1.04 (0.97, 1.11)	1.07 (0.99, 1.14)	1.06 (0.97, 1.17)
71-75	Ref	1.02 (0.96, 1.08)	1.08 (1.02, 1.16)	1.16 (1.07, 1.27)
75-80	Ref	1.10 (1.04, 1.17)	1.11 (1.04, 1.18)	1.24 (1.14, 1.35)
81-85	Ref	1.03 (0.97, 1.10)	1.14 (1.07, 1.21)	1.27 (1.17, 1.38)
86+	Ref	1.04 (0.99, 1.10)	1.08 (1.02, 1.13)	1.24 (1.16, 1.32)
Race				
White	Ref	1.05 (1.01, 1.09)	1.10 (1.06, 1.14)	1.20 (1.14, 1.26)
Black	Ref	1.03 (0.96, 1.10)	1.07 (0.99, 1.15)	1.11 (0.98, 1.25)
Hispanic	Ref	0.98 (0.90, 1.07)	0.99 (0.90, 1.09)	1.18 (1.02, 1.35)
Other	Ref	1.07 (0.97, 1.18)	1.09 (0.98, 1.21)	1.10 (0.94, 1.29)
Medicaid				
No	Ref	1.05 (1.01, 1.09)	1.09 (1.05, 1.14)	1.19 (1.13, 1.25)
Yes	Ref	1.03 (0.98, 1.09)	1.08 (1.02, 1.14)	1.15 (1.07, 1.23)
Residence prior to hospitalization				
Community	Ref	1.04 (1.01, 1.08)	1.10 (1.06, 1.14)	1.20 (1.14, 1.26)
Nursing facility or other institutions	Ref	1.04 (0.98, 1.10)	1.05 (0.99, 1.12)	1.09 (0.99, 1.19)
Medical school affiliation				
Major	Ref	1.03 (0.96, 1.11)	1.06 (0.97, 1.16)	1.14 (0.95, 1.37)
Limited	Ref	1.06 (0.98, 1.15)	1.12 (1.03, 1.21)	1.25 (1.12, 1.41)
Graduate	Ref	1.07 (0.35, 3.38)	1.13 (0.39, 3.38)	1.18 (0.22, 6.28)
No affiliation	Ref	1.04 (0.99, 1.09)	1.10 (1.04, 1.15)	1.18 (1.11, 1.25)
Location				
Rural	Ref	0.99 (0.89, 1.09)	1.12 (1.03, 1.22)	1.22 (1.12, 1.32)
Urban	Ref	1.05 (0.99, 1.08)	1.08 (1.04, 1.12)	1.15 (1.08, 1.22)
Type of hospital				
For profit	Ref	1.07 (0.98, 1.16)	1.14 (1.04, 1.24)	1.24 (1.11, 1.38)
Government	Ref	0.92 (0.83, 1.01)	1.09 (0.99, 1.21)	1.20 (1.09, 1.33)
Non-profit	Ref	1.08 (1.03, 1.12)	1.11 (1.06, 1.16)	1.19 (1.12, 1.26)
Hospital quality score				
1	Ref	0.92 (0.69, 1.22)	0.88 (0.64, 1.21)	0.80 (0.48, 1.34)
2	Ref	1.03 (0.94, 1.14)	1.12 (1.02, 1.24)	1.26 (1.09, 1.45)
3	Ref	1.07 (1.02, 1.12)	1.10 (1.04, 1.15)	1.20 (1.12, 1.29)
4	Ref	1.01 (0.95, 1.07)	1.08 (1.01, 1.15)	1.14 (1.04, 1.25)
5	Ref	1.25 (0.99, 1.58)	1.58 (1.20, 2.08)	1.39 (1.04, 1.86)

© 2022 Dang A et al. *JAMA Network Open.*

[a]See footnote to Table e5.
OR, odds ratio; CI, confidence interval.

eTable 8. Odds of mortality in the 30 days after hospital admission from January through March of 2021 by quartile of prevalence of SARS-CoV-2 in the hospital, stratified by characteristics of the admission.[a]

**Admission/hospital characteristic**	SARS-CoV-2 prevalence 0-1.61% OR (95% CI)	SARS-CoV-2 prevalence 1.62-4.55% OR (95% CI)	SARS-CoV-2 prevalence 4.55-8.65% OR (95% CI)	SARS-CoV-2 prevalence >8.65% OR (95% CI)
Disease severity				
Low	Ref	1.00 (0.92, 1.08)	0.99 (0.92, 1.08)	1.02 (0.92, 1.13)
Medium	Ref	1.02 (0.98, 1.07)	1.04 (0.99, 1.09)	1.10 (1.04, 1.16)
High	Ref	1.04 (0.98, 1.10)	1.08 (1.03, 1.15)	1.19 (1.12, 1.26)
Age (Per year)				
<=65	Ref	1.02 (0.94, 1.10)	1.05 (0.97, 1.13)	1.05 (0.95, 1.15)
66-70	Ref	1.02 (0.94, 1.10)	1.01 (0.94, 1.10)	1.11 (1.01, 1.22)
71-75	Ref	1.06 (0.99, 1.14)	1.08 (1.01, 1.16)	1.18 (1.08, 1.29)
75-80	Ref	1.02 (0.95, 1.09)	1.04 (0.97, 1.12)	1.09 (0.99, 1.18)
81-85	Ref	1.04 (0.97, 1.11)	1.07 (0.99, 1.15)	1.18 (1.08, 1.28)
86+	Ref	1.04 (0.99, 1.10)	1.08 (1.02, 1.13)	1.24 (1.16, 1.32)
Race				
White	Ref	1.03 (0.99, 1.08)	1.06 (1.01, 1.10)	1.12 (1.07, 1.18)
Black	Ref	1.02 (0.92, 1.12)	1.07 (0.97, 1.17)	1.18 (1.06, 1.33)
Hispanic	Ref	0.92 (0.82, 1.03)	0.95 (0.85, 1.07)	1.04 (0.91, 1.19)
Other	Ref	1.07 (0.95, 1.20)	1.07 (0.95, 1.20)	1.14 (0.98, 1.31)
Medicaid				
No	Ref	1.03 (0.98, 1.07)	1.04 (0.99, 1.09)	1.13 (1.08, 1.19)
Yes	Ref	1.01 (0.95, 1.08)	1.08 (1.02, 1.15)	1.10 (1.02, 1.18)
Residence prior to hospitalization				
Community	Ref	1.01 (0.97, 1.06)	1.04 (0.99, 1.09)	1.13 (1.08, 1.19)
Nursing facility or other institutions	Ref	1.07 (1.01, 1.15)	1.09 (1.02, 1.17)	1.05 (0.96, 1.15)
Medical school affiliation				
Major	Ref	1.03 (0.94, 1.12)	1.08 (0.98, 1.19)	1.10 (0.94, 1.28)
Limited	Ref	1.06 (0.97, 1.17)	1.09 (0.99, 1.20)	1.14 (1.01, 1.28)
Graduate	Ref	1.21 (0.90, 1.62)	1.29 (0.96, 1.75)	1.21 (0.83, 1.77)
No affiliation	Ref	1.01 (0.95, 1.07)	1.03 (0.97, 1.09)	1.11 (1.05, 1.18)
Location				
Rural	Ref	0.98 (0.89, 1.08)	0.97 (0.88, 1.06)	1.14 (1.04, 1.24)
Urban	Ref	1.03 (0.99, 1.08)	1.07 (1.02, 1.12)	1.10 (1.04, 1.16)
Type of hospital				
For profit	Ref	1.03 (0.91, 1.16)	1.02 (0.91, 1.15)	1.06 (0.94, 1.21)
Government	Ref	0.95 (0.85, 1.06)	1.01 (0.90, 1.13)	1.06 (0.94, 1.18)
Non-profit	Ref	1.03 (0.99, 1.08)	1.06 (1.01, 1.11)	1.15 (1.08, 1.22)
Hospital quality score				
1	Ref	0.98 (0.67, 1.44)	0.94 (0.64, 1.39)	0.89 (0.59, 1.33)
2	Ref	0.97 (0.86, 1.08)	0.99 (0.89, 1.12)	1.09 (0.96, 1.24)

3	Ref	1.01 (0.95, 1.07)	1.04 (0.98, 1.10)	1.16 (1.08, 1.24)
4	Ref	1.08 (0.99, 1.17)	1.10 (1.01, 1.19)	1.18 (1.07, 1.30)
5	Ref	1.10 (0.86, 1.40)	1.10 (0.84, 1.44)	1.15 (0.86, 1.53)

[a] See footnote to Table e-5.
OR, odds ratio; CI, confidence interval.

eTable 9. Odds of mortality in the 30 days after hospital admission from April through June of 2021 by quartile of prevalence of SARS-CoV-2 in the hospital, stratified by characteristics of the admission.[a]

**Admission/hospital characteristic**	SARS-CoV-2 prevalence 0% OR (95% CI)	SARS-CoV-2 prevalence 0.01-2.12% OR (95% CI)	SARS-CoV-2 prevalence >=2.13% OR (95% CI)
Disease severity			
Low	Ref	0.99 (0.89, 1.09)	1.02 (0.91, 1.15)
Medium	Ref	0.97 (0.92, 1.03)	1.05 (0.99, 1.12)
High	Ref	0.96 (0.89, 1.02)	1.01 (0.93, 1.08)
Age (Per year)			
<=65	Ref	1.03 (0.93, 1.14)	1.04 (0.93, 1.17)
66-70	Ref	0.98 (0.89, 1.09)	1.01 (0.90, 1.13)
71-75	Ref	1.03 (0.94, 1.14)	1.08 (0.96, 1.20)
75-80	Ref	1.02 (0.93, 1.12)	1.08 (0.98, 1.20)
81-85	Ref	0.89 (0.81, 0.97)	0.96 (0.87, 1.06)
86+	Ref	0.93 (0.87, 1.01)	1.03 (0.95, 1.11)
Race			
White	Ref	0.97 (0.92, 1.02)	1.03 (0.98, 1.09)
Black	Ref	0.97 (0.85, 1.11)	1.01 (0.86, 1.16)
Hispanic	Ref	0.85 (0.76, 0.95)	1.01 (0.87, 1.16)
Other	Ref	1.14 (0.99, 1.31)	1.07 (0.90, 1.28)
Medicaid			
No	Ref	0.97 (0.92, 1.02)	1.04 (0.98, 1.10)
Yes	Ref	0.97 (0.91, 1.05)	0.99 (0.92, 1.08)
Residence prior to hospitalization			
Community	Ref	0.94 (0.90, 0.99)	1.01 (0.96, 1.07)
Nursing facility or other institutions	Ref	1.10 (0.99, 1.20)	1.11 (1.00, 1.24)
Medical school affiliation			
Major	Ref	0.96 (0.81, 1.14)	1.01 (0.83, 1.21)
Limited	Ref	1.18 (1.04, 1.35)	1.32 (1.15, 1.52)
Graduate	Ref	1.07 (0.69, 1.68)	1.15 (0.70, 1.90)
No affiliation	Ref	0.95 (0.90, 1.01)	1.01 (0.95, 1.07)
Location			
Rural	Ref	0.96 (0.89, 1.04)	0.96 (0.89, 1.04)
Urban	Ref	0.98 (0.93, 1.04)	1.08 (1.01, 1.15)
Type of hospital			
For profit	Ref	1.03 (0.92, 1.15)	1.01 (0.88, 1.14)
Government	Ref	0.93 (0.83, 1.04)	0.97 (0.87, 1.09)
Non-profit	Ref	0.96 (0.90, 1.02)	1.05 (0.98, 1.12)
Hospital quality score			
1	Ref	0.87 (0.58, 1.32)	0.96 (0.62, 1.50)

2	Ref	1.07 (0.94, 1.21)	1.03 (0.90, 1.19)
3	Ref	1.04 (0.96, 1.12)	1.11 (1.02, 1.21)
4	Ref	0.89 (0.81, 0.98)	0.97 (0.88, 1.08)
5	Ref	1.02 (0.81, 1.29)	0.84 (0.64, 1.10)

[a] See footnote to Table e-5.
OR, odds ratio; CI, confidence interval.

eTable 10. Odds of mortality in the 30 days after hospital admission from July through September of 2021 by quartile of prevalence of SARS-CoV-2 in the hospital, stratified by characteristics of the admission.[a]

**Admission/hospital characteristic**	SARS-CoV-2 prevalence 0-1.27% OR (95% CI)	SARS-CoV-2 prevalence 1.28-4.06% OR (95% CI)	SARS-CoV-2 prevalence 4.07-9.09% OR (95% CI)	SARS-CoV-2 prevalence >9.10% OR (95% CI)
Disease severity				
Low	Ref	1.04 (0.97, 1.13)	1.18 (1.09, 1.28)	1.22 (1.10, 1.35)
Medium	Ref	1.02 (0.97, 1.07)	1.11 (1.06, 1.17)	1.30 (1.22, 1.38)
High	Ref	1.03 (0.98, 1.09)	1.12 (1.06, 1.18)	1.24 (1.17, 1.33)
Age (Per year)				
<=65	Ref	1.01 (0.93, 1.08)	1.17 (1.08, 1.26)	1.27 (1.15, 1.40)
66-70	Ref	1.02 (0.94, 1.09)	1.17 (1.09, 1.27)	1.33 (1.20, 1.46)
71-75	Ref	1.07 (0.99, 1.14)	1.18 (1.10, 1.27)	1.26 (1.15, 1.37)
75-80	Ref	1.03 (0.97, 1.10)	1.09 (1.02, 1.17)	1.27 (1.17, 1.39)
81-85	Ref	1.00 (0.93, 1.06)	1.09 (1.01, 1.17)	1.19 (1.09, 1.30)
86+	Ref	1.03 (0.98, 1.09)	1.08 (1.02, 1.15)	1.28 (1.19, 1.37)
Race				
White	Ref	1.03 (0.98, 1.07)	1.11 (1.06, 1.16)	1.26 (1.20, 1.34)
Black	Ref	1.00 (0.92, 1.08)	1.18 (1.08, 1.29)	1.36 (1.21, 1.52)
Hispanic	Ref	1.03 (0.93, 1.13)	1.14 (1.03, 1.27)	1.18 (1.02, 1.37)
Other	Ref	1.05 (0.95, 1.16)	1.26 (1.12, 1.42)	1.16 (0.98, 1.37)
Medicaid				
No	Ref	1.01 (0.97, 1.06)	1.09 (1.04, 1.15)	1.21 (1.15, 1.28)
Yes	Ref	1.06 (1.01, 1.13)	1.21 (1.14, 1.29)	1.43 (1.32, 1.54)
Residence prior to hospitalization				
Community	Ref	1.04 (0.99, 1.08)	1.15 (1.10, 1.21)	1.31 (1.24, 1.39)
Nursing facility or other institutions	Ref	0.98 (0.92, 1.04)	1.00 (0.93, 1.07)	1.00 (0.91, 1.11)
Medical school affiliation				
Major	Ref	1.02 (0.95, 1.11)	1.26 (1.13, 1.41)	1.54 (1.19, 2.01)
Limited	Ref	1.07 (0.98, 1.17)	1.20 (1.10, 1.31)	1.49 (1.31, 1.69)
Graduate	Ref	1.10 (0.77, 1.58)	1.23 (0.83, 1.83)	1.28 (0.65, 2.53)
No affiliation	Ref	1.03 (0.97, 1.09)	1.10 (1.04, 1.17)	1.23 (1.16, 1.32)
Location				
Rural	Ref	1.04 (0.92, 1.17)	1.12 (1.01, 1.25)	1.26 (1.14, 1.40)
Urban	Ref	1.02 (0.98, 1.07)	1.12 (1.07, 1.18)	1.27 (1.19, 1.36)
Type of hospital				
For profit	Ref	1.19 (1.06, 1.34)	1.31 (1.16, 1.48)	1.37 (1.18, 1.58)
Government	Ref	1.02 (0.90, 1.16)	1.10 (0.97, 1.25)	1.15 (1.01, 1.31)

© 2022 Dang A et al. *JAMA Network Open.*

Non-profit	Ref	1.01 (0.97, 1.06)	1.11 (1.05, 1.17)	1.31 (1.23, 1.40)
Hospital quality score				
1	Ref	0.97 (0.73, 1.31)	1.01 (0.69, 1.50)	1.23 (0.79, 1.90)
2	Ref	1.12 (1.01, 1.24)	1.25 (1.11, 1.40)	1.57 (1.35, 1.81)
3	Ref	1.01 (0.95, 1.06)	1.08 (1.01, 1.15)	1.24 (1.14, 1.35)
4	Ref	1.10 (1.02, 1.18)	1.24 (1.15, 1.34)	1.39 (1.27, 1.53)
5	Ref	1.12 (0.88, 1.43)	1.45 (1.10, 1.90)	1.35 (0.99, 1.83)

[a] See footnote to Table e-5.
OR, odds ratio; CI, confidence interval.

eTable 11. Unadjusted rates and adjusted odds of mortality after hospitalization for non-SARS-CoV-2 medical admissions in 2020 vs. 2019, using different methods to exclude SARS-CoV-2 cases.

	Unadjusted rate Q_2 2020 vs. Q_2 2019	Mortality OR (95% CI) for Q_2 2020 vs. Q_2 2019
1. Excluding hospitalizations with a SARS-CoV-2 admission diagnosis or the first two discharge diagnoses. (Method used in our analyses)	11.44% vs. 9.18%	1.20 (1.19, 1.21)
2. Excluding hospitalizations with a SARS-CoV-2 admission diagnosis or any diagnosis of SARS-CoV-2 in any position in the discharge diagnoses.	11.44% vs 9.18%	1.20 (1.19-1.21)
3. Exclusion in #2 plus any SARS-CoV-2 diagnosis during hospitalization or prior to hospitalization.	11.19% vs 9.18%	1.19 (1.17, 1.20)
4. Exclusions in #2 and #3 plus any SARS-CoV-2 diagnosis in the 30 days after hospital discharge.	11.18% vs 9.18%	1.19 (1.17, 1.20)

OR, odds ratio; CI, confidence interval.

© 2022 Dang A et al. *JAMA Network Open.*
